# Beyond SrTiO_3_: Emerging Perovskite Photocatalysts for Solar Water Splitting

**DOI:** 10.3390/ma19173635

**Published:** 2026-08-26

**Authors:** Farzaneh Talebkeikhah, Hojjatollah Ahmadi Rad, Mohammad Hasan Faghani, Morteza Zokaeian

**Affiliations:** 1 Center for Energy and Environmental Sciences, Paul Scherrer Institute (PSI), 5232 Villigen, Switzerland; 2 School of Chemical Engineering, College of Engineering, University of Tehran, Tehran 57131-14399, Iran; 3 Department of Chemical Engineering, Amirkabir University of Technology (Tehran Polytechnic), Tehran 15875-4413, Iran; hasanfaghani@aut.ac.ir

**Keywords:** perovskites, photocatalysis, water splitting, SrTiO_3_

## Abstract

Photocatalysis has emerged as a promising strategy for converting solar energy into chemical fuels, offering a sustainable route for hydrogen production through water splitting. While solar energy has been successfully utilized for electricity generation via photovoltaic technologies, extending its use to direct chemical fuel production, analogous to natural photosynthesis, remains a major challenge. A significant breakthrough was recently reported by Takata et al., who demonstrated that Al-doped SrTiO_3_ loaded with Rh/Cr_2_O_3_ & CoOOH cocatalysts can achieve near-unity quantum efficiency for photocatalytic water splitting under ultraviolet irradiation at wavelengths around 350 nm. Motivated by this progress, this review surveys perovskite-based photocatalysts for water splitting with the aim of identifying materials beyond SrTiO_3_ having the potential to exhibit similar or improved performance. The fundamentals of photocatalytic water splitting and the properties of SrTiO_3_ and other perovskite families, including oxide, oxynitride, Aurivillius, Dion–Jacobson, and Ruddlesden–Popper perovskites, are discussed. Notably promising perovskite materials with potential for achieving high efficiencies and improved solar-light utilization are highlighted.

## 1. Introduction

Photocatalysis has emerged as a powerful strategy for utilizing solar energy to drive chemical transformations, offering promising opportunities for renewable fuel production, environmental remediation, and sustainable chemical synthesis [1]. Among these applications, the conversion of solar energy into chemical fuels is particularly attractive because it enables the storage of intermittent solar energy in the form of stable energy carriers. Hydrogen produced from water using sunlight represents one of the most compelling examples of this concept and is widely regarded as a key component of future carbon-neutral energy systems [2]. Over the past decades, substantial progress has been achieved in harnessing solar energy for electricity generation through photovoltaic technologies. The rapid deployment of large-scale solar power plants worldwide demonstrates the technological maturity and scalability of solar electricity production. Notable examples include the 550 MW Topaz Solar Farm (USA), 800 MW Copper Mountain Solar Facility (USA) [3], 605 MW Witznitz Solar Farm (Germany), 1500 MW Tengger Desert Solar Park (China) [4], 1650 MW Benban Solar Park (Egypt) [5], 1300 MW Karapınar Solar Power Plant (Turkey) [6], 1100 MW Noor Abu Dhabi (United Arab Emirates), 1000 MW Kurnool Ultra Mega Solar Park (India), and the 2000 MW Pavagada Solar Park (India) [7,8,9]. These installations highlight the enormous potential of solar energy as a large-scale and sustainable electricity source. In contrast, the direct conversion of solar energy into chemical fuels remains far less developed. Achieving efficient solar-driven chemical production presents significant challenges due to the complexity of the underlying photophysical and catalytic processes. Photocatalysis offers a promising pathway to address these challenges by enabling light-driven chemical reactions that convert solar energy directly into chemical bonds.

The modern field of photocatalysis originated from the pioneering discovery of photoelectrochemical water splitting on TiO_2_ electrode by Fujishima and Honda in 1972, which demonstrated that light irradiation could drive the splitting of water into hydrogen and oxygen [10]. This landmark discovery initiated extensive research into semiconductor photocatalysis, leading to a wide range of applications including pollutant degradation [11], methane activation [12], alkane functionalization [13], CO_2_ reduction [14,15], N_2_ fixation [16], decarboxylation reactions [17], and hydrodeoxygenation processes [18]. Among these reactions, photocatalytic water splitting has attracted particular attention because it directly links solar energy conversion with sustainable hydrogen production. Hydrogen is a clean and energy-dense fuel whose combustion produces only water, making it an attractive alternative to fossil fuels. In addition to its technological relevance, water splitting also serves as a benchmark reaction for evaluating photocatalytic materials, as it allows systematic investigation of fundamental parameters such as light absorption, charge carrier generation and separation, and surface reaction kinetics [19].

Despite significant research efforts, the efficiency of photocatalytic water splitting remains considerably lower than that achieved by alternative hydrogen production technologies. For practical solar hydrogen generation, a solar-to-hydrogen (STH) efficiency of approximately 10% is generally considered the minimum threshold for economic viability. However, most particulate photocatalyst systems currently exhibit STH efficiencies below 1%, with typical values ranging from 0.01–0.5% under simulated solar irradiation [20]. Only a limited number of highly optimized systems, such as modified Al-doped SrTiO_3_ photocatalysts combined with cocatalysts, have approached efficiencies of 1–2% [9,21,22,23]. In contrast, photovoltaic-powered electrolyzers can achieve overall STH efficiencies of approximately 15–20%, while advanced photoelectrochemical tandem systems have reported efficiencies exceeding 10–19% under laboratory conditions [24]. This disparity highlights the key limitations of current photocatalytic systems, including insufficient visible-light absorption, rapid recombination of photogenerated charge carriers, and sluggish surface reaction kinetics. An efficient photocatalyst for overall water splitting must therefore satisfy several critical physicochemical requirements.

A major breakthrough in this field was recently reported by Takata et al. [21], who demonstrated nearly unity quantum efficiency for overall photocatalytic water splitting using an Al-doped SrTiO_3_ photocatalyst. In this system, aluminum was incorporated into the SrTiO_3_ lattice at concentrations below 1 atom% relative to Ti. Furthermore, cocatalyst nanoparticles were selectively deposited on different crystallographic facets via photodeposition, with Ru/Cr_2_O_3_ clusters deposited on electron-collecting {100} facets and CoOOH deposited on hole-collecting {110} facets. This facet-engineered architecture enabled highly efficient spatial separation of photogenerated charge carriers, resulting in an external quantum efficiency of up to 96% at wavelengths between 350 and 360 nm. These results highlight the potential of carefully engineered perovskite photocatalysts to achieve extremely high efficiencies in photocatalytic water splitting. Motivated by this finding, perovskite materials have the potential to be one of the most promising classes of photocatalysts for solar-driven water splitting. The structural flexibility of perovskites allows extensive tuning of their electronic structure, optical absorption properties, and charge transport characteristics through compositional modification, doping, and structural engineering [25].

In this review, we focus on perovskite-based photocatalysts for water splitting, with the goal of identifying the potential materials that may achieve higher efficiencies and improved solar-light utilization. The concept underlying this review originated from the observation in the pioneering work of Takata et al. on SrTiO_3_. As mentioned before, Takata and co-workers demonstrated that exceptional photocatalytic performance can be achieved not through the discovery of a new material, but through the rational modification of an existing one. Their strategy combined three key elements: low-level Al substitution (≈1 at.%) at the Ti site, molten-salt synthesis to produce highly crystalline particles with minimal defects, and photodeposition of optimized cocatalysts. The synergy between these modifications enabled SrTiO_3_ to exhibit photocatalytic water splitting with an apparent quantum efficiency approaching unity under ultraviolet irradiation. Motivated by this success, we first discuss the fundamental principles of photocatalysis, including band-gap engineering, band-edge alignment, cocatalysts, doping effects and the role of sacrificial agents. Subsequently, the structural characteristics and classifications of perovskite materials are introduced. The extensively studied SrTiO_3_ system is then examined as a benchmark perovskite photocatalyst. Finally, we explore other perovskite families including simple & double oxide perovskites, oxynitrides, Dion–Jacobson, Aurivillius, and Ruddlesden–Popper phases, to evaluate whether they could benefit from the same materials-engineering strategy that enabled the exceptional performance of SrTiO_3_. This review seeks to answer the following question: Are there perovskite photocatalysts beyond SrTiO_3_ with electronic structures intrinsically suitable for overall water splitting that, through the application of similar materials-engineering principles (including simultaneously improved crystallinity, optimized doping strategies and rational cocatalyst deposition), could achieve photocatalytic performances comparable to the near-unity quantum efficiency demonstrated by SrTiO_3_ system? By addressing this question, the review suggests that, rather than focusing on the discovery of new perovskite compositions, research should concentrate on engineering the already proven and promised perovskites. The novelty of this review lies in its exclusive focus on photocatalytic water splitting and its approach of evaluating perovskite photocatalysts from the same perspective as the SrTiO3-based benchmark [21]. Many existing reviews [26,27,28] focus on visible-light-active photocatalysts covering all applications including environmental applications, which are generally less challenging than water splitting and require less stringent material engineering. Other reviews primarily address perovskites for semiconductor and electronic applications [29,30]. Although a few reviews [31,32] focus only on water splitting, they typically cover both photocatalytic and photoelectrochemical systems rather than concentrating solely on photocatalytic water splitting.

## 2. Fundamental Principles of Photocatalysis

For photocatalytic water splitting to occur, a minimum thermodynamic potential of 1.23 V is required, corresponding to a Gibbs free energy change of 237.13 kJ mol^−1^ for water splitting reaction as shown in Equation (1).

(1) $$H_{2}O\to H_{2}+1/2O_{2}$$

In photocatalysis, this driving force is generated by photoexcitation of electrons across the band gap of the photocatalyst, producing electron–hole pairs capable of driving reduction and oxidation reactions. In principle, a semiconductor with a band gap larger than 1.23 eV could provide the minimum energy required for water splitting. However, practical systems require additional overpotential to overcome kinetic barriers associated with the multielectron hydrogen and oxygen evolution reactions. Consequently, photocatalysts for solar-driven water splitting must typically exhibit band gaps in the range of 1.8–2.4 eV, allowing visible-light absorption while maintaining sufficient redox driving force. Although an appropriate band gap (typically 1.8–2.4 eV) enables visible-light absorption, it is only one of several essential requirements for efficient overall photocatalytic water splitting. Many promising photocatalysts still exhibit poor overall water-splitting performance because multiple thermodynamic, kinetic, and structural factors must be simultaneously optimized. First, a suitable band gap does not necessarily ensure proper band-edge alignment. The conduction band minimum (CBM) must be more negative than the $H^{+}$/$H_{2}$ reduction potential (0 V vs. NHE), while the valence band maximum (VBM) must be more positive than the $H_{2}O$/$O_{2}$ oxidation potential (1.23 V vs. NHE). Many perovskites possess favorable band gaps but insufficient redox driving force for either hydrogen or oxygen evolution, making overall water splitting thermodynamically infeasible [15,33]. These perovskites have demonstrated only hydrogen or oxygen evolution in the presence of sacrificial reagents rather than unbiased overall water splitting, indicating that efficient charge separation and balanced surface redox reactions have not yet been achieved simultaneously [33]. Moreover, rapid recombination of photogenerated electron–hole pairs remains one of the primary causes of efficiency loss. Bulk defects, surface trap states, and limited carrier mobility significantly shorten charge-carrier lifetimes, preventing electrons and holes from reaching active surface sites before recombination [34]. Consequently, defect engineering, heterojunction construction, and cocatalyst loading have become essential strategies to promote charge separation and suppress recombination. Surface reaction kinetics also represent a major limitation. While the hydrogen evolution reaction involves a two-electron process, oxygen evolution is a kinetically demanding four-electron reaction requiring multiple reaction intermediates and high activation energy. Therefore, even photocatalysts with suitable electronic structures still require cocatalysts such as Pt, Rh/Cr_2_O_3_, CoO_x_, or RuO_2_ to accelerate interfacial charge transfer and reduce reaction overpotentials [35]. Furthermore, many visible-light-responsive perovskites suffer from photocorrosion, structural instability, or defect accumulation under prolonged irradiation, resulting in gradual activity loss. Although oxide perovskites generally exhibit better chemical stability than halide perovskites, long-term durability under practical operating conditions remains an important challenge [15,36]. Therefore, the only major challenge is no longer just the suitable band gaps, but the development of integrated material-design strategies that simultaneously optimize band-edge alignment, defect chemistry, charge-carrier dynamics, cocatalyst architecture, surface reaction kinetics, and long-term stability.

The electronic structure of photocatalysts is typically determined using a combination of spectroscopic, electrochemical, and theoretical methods. The band gap energy is commonly estimated using UV–Vis diffuse reflectance spectroscopy (DRS). Then it is analyzed using Tauc plots, which enable determination of direct or indirect band gaps [37]. The valence band position can be measured using X-ray photoelectron spectroscopy (XPS) or ultraviolet photoelectron spectroscopy (UPS). UPS provides particularly accurate information regarding the valence band maximum relative to the Fermi level, whereas XPS valence-band spectra can also be used to estimate the energy difference between the valence band edge and the Fermi level [38,39]. The conduction band position is often estimated using Mott–Schottky analysis derived from electrochemical impedance measurements. From the Mott–Schottky plot, the flat-band potential can be obtained, which is commonly approximated as the conduction band edge for n-type semiconductors. Combining this value with the experimentally determined band gap enables estimation of the valence band edge [40,41].

In addition to experimental techniques, density functional theory (DFT) calculations are widely used to investigate electronic structures. DFT enables prediction of band structures, density of states (DOS), and band-edge positions, while also providing insight into orbital contributions, charge distribution, and the effects of doping or defects. When needed, the description of the electronic structure and band gaps can be further improved using methods such as hybrid functionals (e.g., HSE06) or many-body perturbation methods (e.g., GW) [42]. In some cases, band-edge positions can also be estimated using empirical electronegativity-based models:

E_CB_ = x − E_e_ − 1/2 E_g_, (2)

E_VB_ = E_CB_ + E_g_, (3)

where x is the absolute electronegativity of the semiconductor, $E_{e}$ represents the free-electron energy on the hydrogen scale (≈4.5 eV), and $E_{g}$ is the band gap energy. Although this method provides approximate values, it is frequently used for preliminary evaluation of photocatalyst suitability [43].

Beyond thermodynamic considerations, charge separation efficiency represents a major challenge in photocatalysis. Following photoexcitation, electrons and holes must migrate to the catalyst surface before recombining. However, recombination often occurs on ultrafast timescales. For instance, studies on TiO_2_ have shown that nearly 60% of photogenerated electron–hole pairs recombine within ~25 ns, while only a small fraction of carriers survive long enough (microsecond timescales) to participate in surface reactions [44]. The efficiency of photocatalytic systems is strongly influenced by the dynamics of charge-carrier generation, transport, and recombination. Understanding these processes requires different and a variety of time-resolved spectroscopic techniques capable of probing carrier lifetimes across multiple timescales [45]. While UV–Vis absorption spectroscopy is used to determine the spectral region in which a material absorbs light, photoluminescence (PL) spectroscopy provides insight into recombination pathways. PL emission arises from radiative recombination of electrons and holes; therefore, strong PL intensity typically indicates high recombination rates, whereas suppressed PL signals suggest more efficient charge separation [46]. Carrier lifetimes are frequently measured using time-resolved photoluminescence (TRPL). In TRPL experiments, short laser pulses (picosecond or nanosecond duration) excite the sample, and the decay of luminescence intensity is monitored over time [47]. Analysis of the decay profile provides quantitative information about recombination kinetics. For ultrafast carrier dynamics, transient absorption spectroscopy (TA) is widely employed. In this pump–probe technique, an ultrafast pump pulse excites the sample, and a delayed probe pulse monitors changes in absorption. By varying the delay time from femtoseconds to nanoseconds, the evolution of excited-state populations and carrier relaxation processes can be resolved [48]. For longer-lived excited states, photoinduced absorption (PIA) spectroscopy is commonly used. PIA measurements operate on microsecond to millisecond timescales and are particularly suitable for detecting long-lived charge carriers [49]. Related pump–probe approaches include diffuse reflectance transient spectroscopy (DRTS) [50,51], which measures transient reflectance changes in strongly scattering materials such as powdered photocatalysts [52]. Carrier transport properties can also be studied using microwave photoconductivity decay (µ-PCD) [53]. In this technique, photoexcited charge carriers temporarily increase the electrical conductivity of the semiconductor, and the decay of photoconductivity is monitored via microwave absorption. Another highly sensitive method is time-correlated single-photon counting (TCSPC), which records photon arrival times with picosecond resolution and enables precise determination of recombination lifetimes [54]. Longer carrier lifetimes are generally favorable for photocatalysis because they increase the probability that photogenerated electrons and holes reach the catalyst surface and participate in redox reactions before recombination. However, a longer lifetime does not necessarily translate into higher photocatalytic activity, as the measured lifetime may also reflect carrier trapping in defect states rather than efficient charge transport. Therefore, lifetime measurements should be interpreted together with complementary techniques such as photocurrent response, electrochemical impedance spectroscopy, etc., to establish the relationship between charge dynamics and photocatalytic performance. These electrochemical techniques further provide valuable information on charge-transfer resistance, carrier transport, and interfacial recombination kinetics. Lower charge-transfer resistance, together with stronger transient photocurrent responses, generally indicates more efficient separation and migration of photogenerated charge carriers [55].

Cocatalysts also play a critical role in photocatalytic water splitting because the photocatalyst alone is often unable to efficiently catalyze multielectron redox reactions. In bare photocatalysts, photogenerated electrons and holes frequently recombine before participating in surface reactions. Depositing cocatalysts on the photocatalyst surface provides active reaction sites and charge-separation centers, facilitating efficient transfer of electrons or holes to reactants. This spatial separation of reduction and oxidation sites significantly suppresses recombination and improves quantum efficiency [56]. For hydrogen evolution, noble metals such as Pt, Rh, and Ru are the widely used cocatalysts. Earth-abundant alternatives including Ni-based catalysts and NiO have also been investigated as HER cocatalysts, particularly for alkaline systems. For oxygen evolution, transition-metal oxides and oxyhydroxides such as RuO_2_, IrO_2_, and RhOOH are commonly employed. These materials efficiently accept photogenerated holes and catalyze water oxidation through multistep proton-coupled electron-transfer processes. Thus, efficient photocatalytic systems rely on the synergistic interaction between the photocatalyst and cocatalysts, which control surface reaction kinetics and improve charge separation [56].

The influence of doping concentration on photocatalytic performance is a critical factor in perovskite design, as it dictates the delicate balance between beneficial charge carrier dynamics, thermodynamic band alignment, and detrimental defect-mediated recombination. It has a non-monotonic (volcano-type) trend, depending on the specific nature of the dopant, the host material and its interaction with the host lattice. The vast majority of doped perovskite systems exhibit a classic non-monotonic, volcano-type relationship where an optimal exists. Chen et al. and Wang et al. demonstrated this for Rh-doped and Ce/N-codoped SrTiO_3_, showing that excessive dopants severely degrade the internal quantum yield [50,51]. This pattern extends to aliovalent codoping systems designed for charge compensation, such as La/Al-codoped, Rh/Sb-codoped, and Cr/Ta-codoped SrTiO_3_ [57,58,59], where an exact stoichiometric balance is required. Deviating from the optimum ratio (e.g., >0.6 mol% La [57], or Sb/Rh ≠ 1.0 [58]) fails to fully suppress undesirable high-valent states (like Rh^4+^ or Cr^6+^) or induces lattice distortion, thereby regenerating recombination centers. Additionally, this non-universal, material-specific optimization extends to perovskite families beyond SrTiO_3_. For example, Jiang et al. showed that Na^+^ doping in SrTiO_3_ exhibits a volcano-type relationship peaking at 4–5 mol% and excessive doping causes severe lattice contraction and defect accumulation [60]. In oxynitride perovskites, Pan et al. and Yang et al. investigated the band engineering of LaTaON_2_ and BaNbO_2_N via Mg^2+^ solid solutions [61,62]. In both systems, increasing the Mg content shifted the valence band maximum to provide a thermodynamic driving force for water oxidation and suppressed intrinsic defects; however, the activity exhibited distinct volcano-type relationships. Beyond these optima, excessive Mg incorporation widened the bandgap and diminished the density of states at the band edges, severely reducing visible light absorption and carrier mobility. Consequently, optimal dopant concentrations are strictly material- and dopant-specific rather than universal. The ideal concentration is governed by a complex interplay of factors, including ionic radii mismatch, the valence state of the dopant, the specific energy position of dopant states, and the host lattice’s tolerance factor and intrinsic defect chemistry. Even within the same oxynitride family, the distinct Mg-doping optima for Ta-based LaTaON_2_ versus Nb-based BaNbO_2_N suggest that the B-site cation’s electronic structure governs how much dopant a lattice can accommodate before defect passivation gives way to band-edge degradation. Therefore, rationalizing doping strategies across oxide, oxynitride, and layered perovskites requires systematic, material-specific optimization.

Sacrificial agents are also widely used in half-reaction photocatalysis to simplify mechanistic studies of water splitting. Because many photocatalysts cannot efficiently drive both HER and OER simultaneously, sacrificial reagents are introduced to selectively consume one type of charge carrier. In hydrogen evolution experiments, organic molecules such as methanol, ethanol, or triethanolamine act as hole scavengers, rapidly reacting with photogenerated holes and suppressing recombination. This process increases the availability of conduction-band electrons for proton reduction. For oxygen evolution studies, oxidizing agents such as AgNO_3_ or FeCl_3_ serve as electron scavengers, enabling photogenerated holes to accumulate and oxidize water. Unlike redox mediators, sacrificial agents are irreversibly consumed during the reaction and are not regenerated. Although these systems do not represent true overall water splitting, they are widely used for catalyst screening and mechanistic investigations [63,64,65,66].

Enhancing photocatalytic performance requires simultaneous improvement of light absorption, charge separation, and surface reaction kinetics. Band-structure engineering through doping, defect engineering, and band-gap tuning can extend visible-light absorption and improve charge transport. Heterojunction architectures, including type-II, Z-scheme, S-scheme, and p–n junctions, introduce internal electric fields that promote spatial separation of electrons and holes. Schottky junctions formed with noble metals act as electron sinks, while plasmonic metals can enhance light harvesting through localized surface plasmon resonance [67,68]. Structural engineering strategies also play a key role. Nanostructured architectures, two-dimensional materials, and mesoporous frameworks reduce carrier diffusion distances and expose highly active surfaces. Conductive carbon materials such as graphene, carbon nanotubes, and MXenes further facilitate rapid electron transport. Other emerging approaches including single-atom catalysis, vacancy engineering, ferroelectric polarization, dye sensitization, and advanced heterostructure design continue to expand the toolbox for achieving efficient solar-driven photocatalysis [69,70,71,72].

## 3. Fundamentals of Perovskite Materials

Perovskites have attracted significant attention for a broad range of applications, including photovoltaic solar cells [73], light-emitting diodes (LEDs), lasers, photodetectors [74], photocatalytic chemical reactions [15,72], photothermal conversion [75], optical sensors [76], photocatalytic environmental remediation [77], and other optical and electronical technologies [78,79]. These diverse applications primarily arise from their outstanding crystal structure and unique optical and electronic properties. These structures constitute a large family of crystalline materials generally described by the typical formula ABX_3_, where A is a relatively large cation occupying a 12-fold coordinated cuboctahedral site, B is a smaller cation in octahedral coordination, and X is an anion. Depending on the anion chemistry, perovskites are commonly categorized into oxide, halide, and chalcogenide systems. Among these, oxide perovskites are the most extensively investigated due to their stability and remarkable diversity of functional properties, including ferroelectricity, magnetism, ionic conductivity, and catalytic activity. The archetypal perovskite structure is derived from the mineral CaTiO_3_. In the ideal cubic configuration, the B-site cation resides at the center of a BX_6_ octahedron, while the A-site cation occupies the cuboctahedral cavity formed by the surrounding anions. In oxide perovskites, the B-site is typically occupied by transition metal cations, whereas the A-site generally hosts alkaline-earth or rare-earth ions. Because the B–O bonds form the rigid octahedral framework, they largely determine the structural stability and electronic properties of the material [80]. At elevated temperatures, many perovskites adopt the ideal cubic structure; however, deviations from this configuration are common. Differences in ionic radii, electronegativity, or bonding preferences often induce octahedral tilting and lattice distortions, lowering the symmetry to tetragonal, orthorhombic, rhombohedral, or monoclinic phases. The structural stability of perovskites can be rationalized using geometric descriptors such as the Goldschmidt tolerance factor and the octahedral factor, while modern density functional theory and data-driven approaches are increasingly used to predict new stable compositions. The perovskite lattice is remarkably flexible and can accommodate a wide variety of elements across the periodic table [81]. Substitution of two different cations at the B-site leads to double perovskites with the formula A_2_BB′X_6_, in which the B-site cations often adopt an ordered rock-salt arrangement [82]. Additionally, interruption of the three-dimensional network by interlayer species gives rise to layered perovskite derivatives, further expanding the structural diversity of this material’s family. From an electronic structure perspective, the valence band in oxide perovskites is primarily composed of hybridized O 2p orbitals with contributions from B-site d orbitals, whereas the conduction band is largely derived from unoccupied B-site d states. This orbital configuration plays a central role in determining optical absorption, charge transport, and photocatalytic behavior [83].

**Figure 1:**
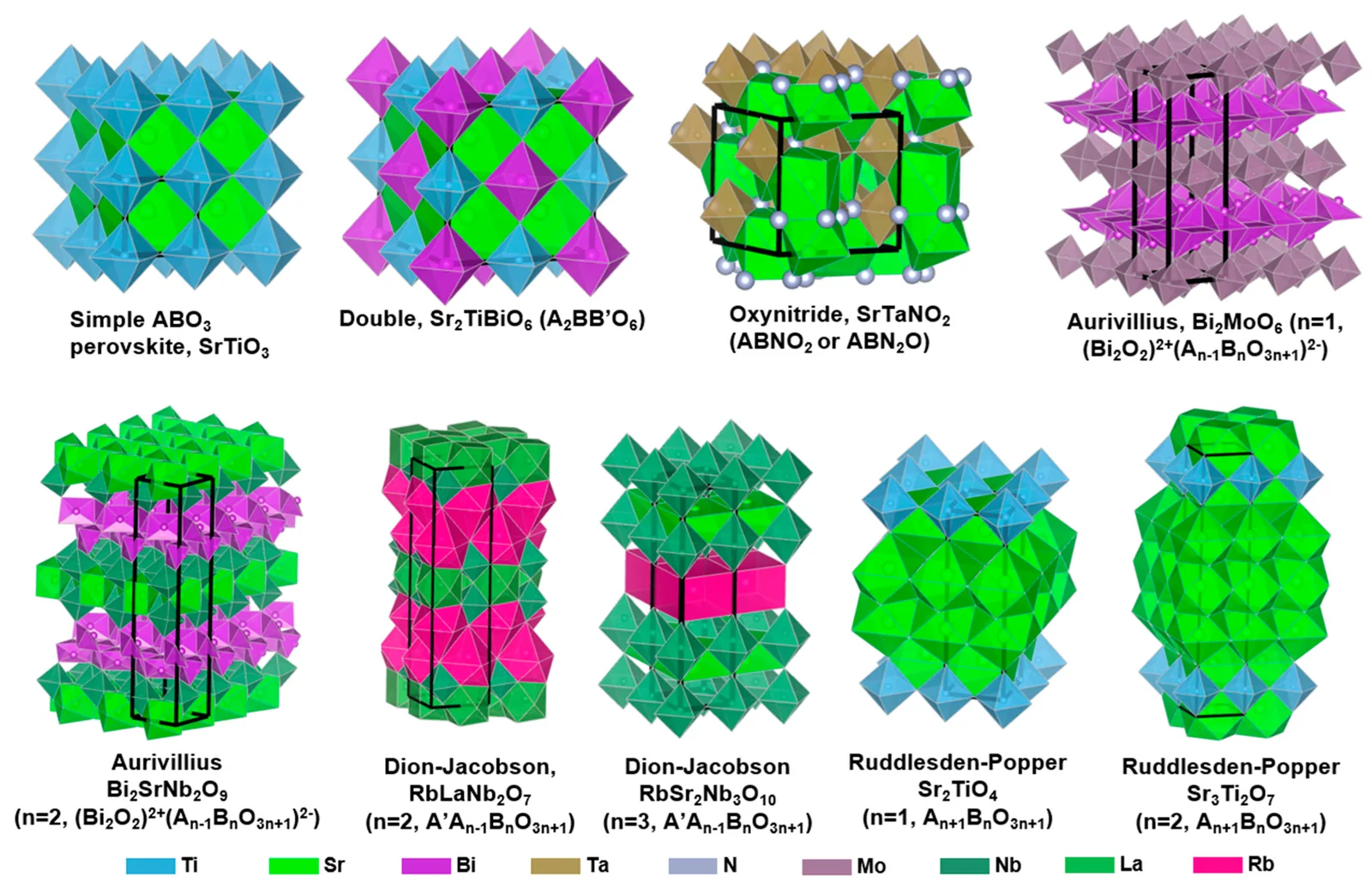
Representative crystal structures of different perovskite families, including simple ABO_3_ perovskites, double perovskites, oxynitrides, Aurivillius phases, Dion–Jacobson phases, and Ruddlesden–Popper layered perovskites. The corresponding CIF files were obtained from the Materials Project database [89,90] and visualized using VESTA software (Version 3.90.3a) [91].

Perovskites can be classified according to cation ordering, dimensionality, crystallographic symmetry, and anion chemistry. From a compositional standpoint, single perovskites contain one type of A-site and B-site cation, whereas double perovskites (A_2_BB′O_6_) feature two distinct B-site cations arranged in an ordered configuration. Such ordering can significantly modify the electronic structure, magnetic interactions, and optical properties through changes in orbital overlap and charge distribution [84]. Another related class is vacancy-ordered perovskites, in which partial B-site vacancies stabilize the structure and influence carrier transport [85]. Dimensionality provides another key classification criterion [86]. Three-dimensional perovskites consist of a continuous network of corner-sharing BX_6_ octahedra extending in all directions. When connectivity is interrupted by interlayer species, layered perovskite structures are formed [87]. Important subclasses include Ruddlesden–Popper (RP), Dion–Jacobson (DJ), and Aurivillius phases, which differ primarily in the nature and charge of the interlayer layers. Ruddlesden–Popper phases typically follow the formula A_n+1_B_n_O_3n+1_ and consist of perovskite slabs separated by rock-salt-type AO layers. In contrast, Dion–Jacobson phases adopt the formula A′A_n−1_B_n_O_3n+1_, in which a single interlayer cation separates adjacent perovskite slabs, resulting in stronger interlayer coupling. Aurivillius phases comprise perovskite-like slabs interleaved with fluorite-like [Bi_2_O_2_]^2+^ layers, giving rise to strong structural anisotropy and frequently stabilizing ferroelectric properties. Another important classification involves anion substitution [88]. Although most perovskites are oxides, partial substitution of oxygen with nitrogen produces perovskite oxynitrides, which retain the structural framework but exhibit stronger covalent bonding, narrower band gaps, and enhanced visible-light absorption. These characteristics make oxynitride perovskites particularly attractive for photocatalytic and photoelectrochemical applications. Representative examples of perovskites from different structural classes are illustrated in Figure 1.

Oxide perovskites can be synthesized through a variety of solid-state and solution-based methods, each offering different levels of control over crystallinity, particle size, morphology, and compositional homogeneity. The solid-state reaction method remains one of the most widely used techniques for preparing bulk perovskites. In this approach, precursor oxides or carbonates are mixed and calcined at high temperatures to promote diffusion and formation of the perovskite phase. Although simple and scalable, this method often yields relatively large particles and limited compositional uniformity [92]. To improve homogeneity and control over particle size, several wet-chemical synthesis routes have been developed. Methods such as sol–gel, citrate, and Pechini synthesis enable molecular-level mixing of metal precursors through chelating agents and polymeric networks, producing highly uniform nanoscale perovskites after calcination. Co-precipitation techniques similarly achieve excellent compositional control by simultaneously precipitating all cations from solution [93]. Other synthesis strategies include hydrothermal and solvothermal methods, which produce well-crystallized nanoparticles under moderate temperatures and elevated pressures with precise control over morphology [94]. Combustion synthesis, based on exothermic reactions between metal salts and organic fuels, enables rapid formation of porous, high-surface-area perovskites that are particularly suitable for catalytic applications [95]. Additional approaches, such as molten-salt synthesis [96], flame spray pyrolysis [97], mechanochemical milling [98] and microwave-assisted synthesis [99] further expand the ability to tailor morphology, crystallinity, and defect concentration.

These synthesis methods also provide versatile platforms for cation doping, a key strategy for tuning the electronic structure, defect chemistry, and catalytic performance of perovskites [100]. In solid-state synthesis, dopants are incorporated during high-temperature diffusion, whereas wet-chemical approaches generally yield more homogeneous dopant distributions due to molecular-level mixing [101]. Hydrothermal and co-precipitation methods allow dopants to enter the lattice during crystal growth, while combustion synthesis often promotes the formation of oxygen vacancies, which can significantly influence catalytic activity. Other techniques, including molten-salt synthesis, mechanochemical doping, ion exchange, and thin-film deposition methods such as pulsed laser deposition, magnetron sputtering, and chemical vapor deposition, allow controlled dopant incorporation during or after perovskite formation. Overall, the wide range of synthesis and doping strategies enables precise control over composition, morphology, defect chemistry, and electronic structure, underpinning the broad applicability of perovskite materials in photocatalysis, electrocatalysis, solid oxide fuel cells, and multifunctional oxide electronics [102].

## 4. Simple ABO_3_ Perovskites

Many perovskites are oxide materials with the general chemical formula ABO_3_, where A is typically an alkaline-earth or rare-earth cation and B is a transition-metal cation. In the ideal cubic structure (space group Pm3m), the B cation is located at the center of the cube and is coordinated by six oxygen atoms forming a BO_6_ octahedron, while the A cation occupies the corners of the cube and is surrounded by twelve oxygen atoms in a cuboctahedral coordination. The structure consists of a three-dimensional framework of corner-sharing BO_6_ octahedra, which creates a highly symmetric lattice with straight B–O–B bond angles of approximately 180°. This high structural symmetry facilitates efficient electronic coupling through the metal–oxygen network and plays an important role in determining the optical and electronic properties of these materials [103].

**Figure 2:**
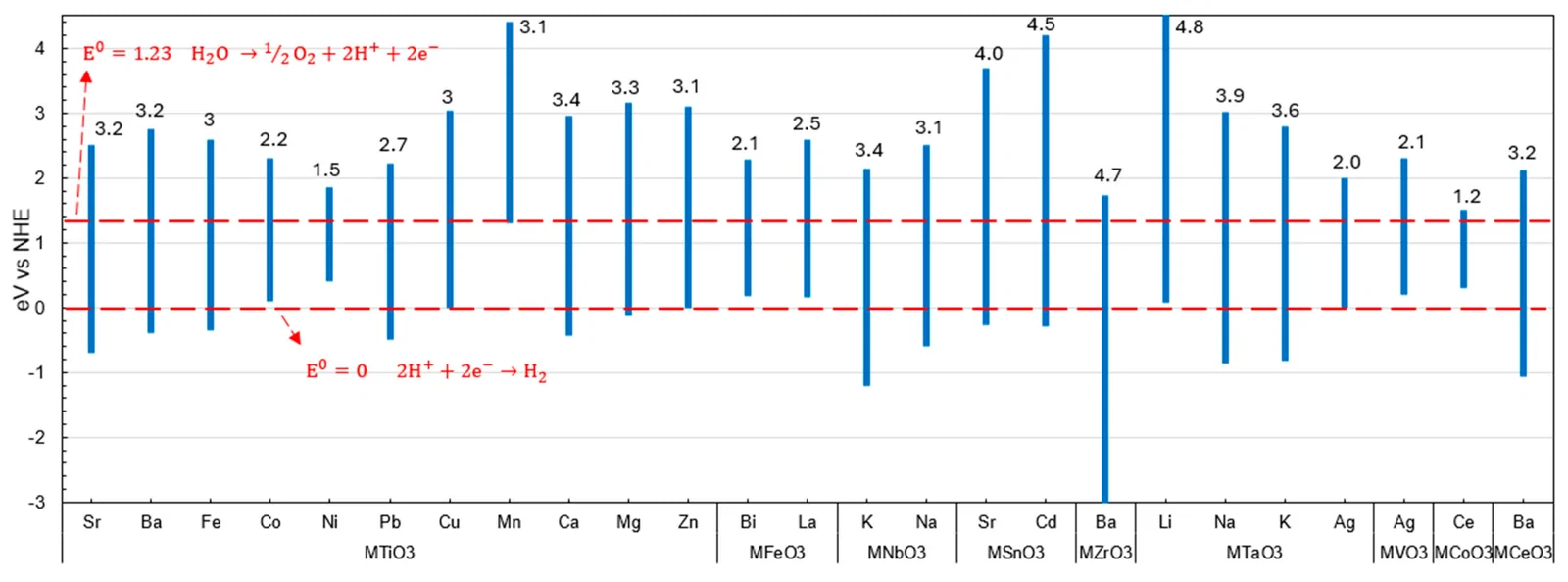
Conduction and valence band edge positions of selected simple ABO_3_-based perovskite oxides relative to the normal hydrogen electrode (NHE), illustrating their suitability for photocatalytic water splitting. The red dashed lines indicate the thermodynamic potentials for hydrogen evolution ($H^{+}$/$H_{2}$, 0 V) and oxygen evolution ($H_{2}O$/$O_{2}$, 1.23 V) [110,111,112,113,114,115,116,117,118,119,120,121,122,123,124,125,126,127,128,129,130,131,132,133,134,135,136].

Because of their structural flexibility, chemical stability, and tunable electronic structure, ABO_3_ perovskites have been widely investigated as photocatalysts for water splitting [28]. Among them, SrTiO_3_ is one of the most extensively studied materials and has served as a model system for understanding photocatalytic water-splitting mechanisms [104,105,106]. Early studies demonstrated that SrTiO_3_, particularly when modified with dopants or suitable cocatalysts, can exhibit high photocatalytic activity and even near-unity quantum efficiency under ultraviolet irradiation. Following this progress, many other oxide perovskites, including BiFeO_3_, LaFeO_3_, and related compounds, have been explored in search of improved visible-light absorption and catalytic performance [107,108]. However, not all oxide perovskites are suitable for photocatalytic water splitting, since the positions of the conduction and valence band must straddle the redox potentials for hydrogen and oxygen evolution. In many cases, the conduction band is not sufficiently negative for hydrogen evolution or the valence band is not positive enough for oxygen evolution, which limits the photocatalytic efficiency despite the favorable crystal structure [109]. Figure 2 demonstrates the conduction and valence band positions of various simple ABO_3_ perovskites, compiled from experimental Mott–Schottky measurements reported in the literature.

Other classes of perovskites lacking reported conduction and valence band positions have also been investigated in the literature. Despite exhibiting band gaps within the visible-light range, these materials have been studied predominantly for environmental applications, such as pollutant degradation, rather than for photocatalytic water splitting. This focus may suggest that their electronic structures or band-edge alignments are not well suited for driving the overall water-splitting reaction. These materials are summarized in Table 1, not as prospective water-splitting candidates, but to provide readers with a comprehensive overview of the current state of the field. Their inclusion is intended to guide future research toward perovskite materials that have already demonstrated water-splitting activity and therefore can be a basis for further optimization through doping, cocatalyst engineering, and other materials-design strategies.

Based on the reported photocatalytic performances (Table 1) and band-edge positions (Figure 2), PbTiO_3_, among the ABO_3_-type perovskites, possesses a narrower band gap than SrTiO_3_ and therefore appears to be a promising candidate for efficient solar water splitting. Given its narrower band gap, PbTiO_3_ seems to have the potential to extend light absorption closer to the visible region, making it an attractive material for further investigation. To evaluate this possibility, we first focus on SrTiO_3_ as a benchmark system to understand the origins of its exceptional photocatalytic performance and subsequently assess whether PbTiO_3_ can be engineered to achieve similar behavior.

Al-doped SrTiO_3_ with cocatalyst has been widely recognized as one of the most efficient photocatalysts for overall water splitting, with reports demonstrating quantum efficiencies approaching unity under ultraviolet irradiation when appropriate cocatalysts and dopants are employed [21]. This remarkable performance has made this photocatalyst a benchmark material in the field of photocatalytic hydrogen production and a model system for understanding the fundamental processes governing charge generation, separation, and surface redox reactions in oxide photocatalysts. In particular, strategies such as Al doping and surface cocatalyst loading (e.g., Rh/Cr_2_O_3_ and CoOOH) have been shown to significantly suppress charge recombination and enhance the efficiency of water-splitting reactions. A detailed understanding of the mechanisms underlying the high activity of modified SrTiO_3_ provides essential design principles for the development of next-generation photocatalysts. Following this detailed analysis of SrTiO_3_, this review then investigates PbTiO_3_, which, among the ABO_3_-type perovskites, has been shown to possess suitable band-edge positions for overall water splitting. PbTiO_3_ is then critically analyzed to evaluate its potential for overall water splitting and to assess whether, after appropriate material-specific modifications, including optimized doping and cocatalyst deposition, it could become a promising candidate for achieving a near-unity quantum efficiency. It must be emphasized that, in this context, approaching the high quantum efficiency demonstrated for optimized SrTiO_3_ represents a hypothetical research objective rather than an expected outcome based on the currently available evidence. Although ABO_3_-type perovskites have been used widely for photocatalysis, their principal limitation is the wide band gap, which restricts light absorption predominantly to the ultraviolet region and significantly limits solar energy utilization [27]. While extensive efforts involving elemental doping, defect engineering, and cocatalyst deposition have substantially improved charge separation and reaction kinetics, these strategies cannot fundamentally overcome the intrinsic limitation of insufficient visible-light absorption [86,104,195]. Among all the reported perovskites, PbTiO_3_ is a relative exception because it combines visible-light absorption with suitable band-edge positions for overall water splitting.

### 4.1. SrTiO_3_

SrTiO_3_ crystallizes in an ideal cubic perovskite structure with a lattice constant of approximately 3.905 Å. According to Shannon ionic radii, Sr^2+^ (1.44 Å, coordination number 12), Ti^4+^ (0.605 Å, coordination number 6), and O^2−^ (1.40 Å) yield a Goldschmidt tolerance factor close to unity, indicating minimal lattice distortion and a highly symmetric crystal structure. This structural stability, combined with favorable band-edge positions relative to the water redox potentials and excellent resistance to photocorrosion, has made SrTiO_3_ suitable for water-splitting photocatalysis. A major breakthrough in the field was reported by Takata and co-workers [21], who demonstrated that Al-doped SrTiO_3_ loaded with suitable cocatalysts can achieve nearly unity apparent quantum efficiency for overall water splitting under ultraviolet irradiation around 350 nm. In this system, highly crystalline SrTiO_3_ particles were synthesized through a molten-salt method with approximately 1 at.% Al substitution at the Ti site and calcined at 1150 °C. Sequential photodeposition of cocatalysts Rh, Cr_2_O_3_, and CoOOH resulted in spatially separated reduction and oxidation sites on different crystal facets. Microscopic analyses revealed that Rh/Cr_2_O_3_ nanoparticles preferentially deposited on {100} facets, whereas CoOOH accumulated on {110} facets. This anisotropic distribution originates from internal electric fields generated by work-function differences between facets, which drive directional charge migration within individual particles. Electrons preferentially accumulate on the {100} facets while holes migrate to {110} facets, effectively separating hydrogen and oxygen evolution reactions. Simulations suggest that a work-function difference of approximately 0.2 eV between facets is sufficient to induce strong anisotropic charge separation. This facet-dependent charge rectification suppresses recombination and enables nearly complete utilization of photogenerated carriers, leading to apparent quantum efficiencies approaching 100% [21]. Surface reconstruction induced by facet engineering has also been shown to play a decisive role in improving the photocatalytic performance of SrTiO_3_. Sokolov et al. demonstrated that introducing nano step-shaped facets parallel to the (100) and (110) planes significantly enhanced overall water-splitting performance compared with conventional octadecahedral SrTiO_3_. The reconstructed step-shaped surfaces were enriched with Sr and contained abundant oxygen vacancies and Ti deficiencies, leading to a modified surface electronic structure. The coexistence of (100) and (110) facets promoted directional charge separation through facet-dependent band bending, where photogenerated electrons and holes preferentially migrated to different crystal facets, thereby suppressing charge recombination. Moreover, the high density of oxygen vacancies and Ti^3+^ species increased the availability of active reaction sites, strengthened reactant adsorption, and facilitated interfacial charge transfer. Consequently, the synergistic effects of facet engineering, surface atomic reconstruction, and defect formation resulted in substantially improved photocatalytic hydrogen evolution [196]. The spatial arrangement of exposed crystal facets can also govern charge-carrier dynamics in SrTiO_3_ photocatalysts. Wang et al. demonstrated that decahedral SrTiO_3_ crystals enclosed by {100} and {111} facets exhibited superior photocatalytic hydrogen evolution compared with cubic particles exposing predominantly {100} facets. The enhanced activity originated from the anisotropic distribution of photogenerated charge carriers, where electrons preferentially accumulated on the {100} facets while holes migrated to the {111} facets, thereby suppressing electron–hole recombination. In addition, selective photodeposition experiments confirmed that reduction and oxidation cocatalysts were preferentially deposited on different facets, providing spatially separated active sites for the hydrogen and oxygen evolution reactions. This facet-dependent charge separation, together with the optimized surface atomic configuration of the decahedral morphology, significantly accelerated interfacial redox reactions and improved the overall efficiency of photocatalytic water splitting [197]. A computational investigation of SrTiO_3_ revealed that photocatalytic activity is highly sensitive to surface atomic configuration, particularly the presence of stepped structures rather than ideal flat facets. The study compared flat {001} surfaces with stepped surfaces representing multifaceted SrTiO_3_ nanocrystals containing ridge, slope, and gully regions. The stepped morphology, particularly the ridge sites, exhibited significantly stronger water adsorption than the flat {001} surface, favouring only dissociative adsorption with spontaneous oxygen vacancy formation. Moreover, adsorption energies of key OER intermediates (HO*, O*, and HOO*) were generally more favourable on the ridge region, indicating enhanced catalytic activity. In contrast, the slope region behaved similarly to the flat {001} facet, where both molecular and dissociative water adsorption were possible. These findings suggest that the superior photocatalytic performance of multifaceted SrTiO_3_ originates not merely from exposing additional facets but from the creation of stepped atomic configurations that modify the local electronic environment, promote oxygen-vacancy formation, strengthen adsorption of reactants and intermediates, and facilitate charge separation across surfaces with different orientations [196].

Extensive studies have revealed that the enhanced photocatalytic activity of SrTiO_3_ is closely related to defect chemistry and surface structure, and facet engineering. A primary mechanism of surface reconstruction involves the thermodynamic stabilization of low-valence cations and oxygen vacancies (VO) under reducing or photoexcited conditions. Thermal treatments in the presence of reducing agents such as NaBH_4_ induce the formation of a disordered, defect-rich overlayer on SrTiO_3_ nanocrystals [195]. Liu et al. demonstrated that this surface reconstruction introduces abundant VO and Ti^3+^/Ti^2+^ sites, which act as electron donors, upshift the Fermi level, and induce downward band bending, thereby enhancing interfacial charge transfer and extending light absorption into the visible/infrared regions, while simultaneously tuning the H_2_:O_2_ evolution ratio [195]. Notably, this benefit is non-monotonic with respect to defect concentration: as the hydrogenation temperature and correspondingly the oxygen-vacancy concentration were increased from 320 to 350 °C, the H_2_ evolution rate peaked at 202.8 μmol h^−1^ (~1.5× that of pristine SrTiO_3_), coinciding with the lowest photo-luminescence (PL) intensity and thus the most efficient charge separation. However, further increasing the treatment temperature to 380 °C (11.7% VO concentration) caused the H_2_ evolution rate to fall back to 182.1 μmol h^−1^, accompanied by a marked increase in PL intensity. This decline indicates that once the vacancy concentration exceeds an optimal threshold, the excess VO sites switch from acting as beneficial electron donors to acting as charge-recombination centers on the surface, annihilating photogenerated electron–hole pairs rather than facilitating their separation. This establishes that surface defect engineering carries an intrinsic volcano-type optimum rather than a monotonic more defects, better performance relationship. Complementing these bulk-defect studies, Lehner et al. highlighted the environmental stability of the p(6 × 2) surface reconstruction on SrTiO_3_ {001}, where pyramidal-coordinated Ti^3+^ and square-planar Ti^3+^ configurations form stable, metallic quasi-particle states at the Fermi level that persist even under near-ambient pressures of water vapor [198]. This defect-mediated reconstruction is not limited to intrinsic host cations; heteroatom doping also triggers dynamic surface relaxation. Gherca et al. revealed that in monophasic Rh-doped SrTiO_3_, a reversible Rh^3+^/Rh^4+^ redox interplay, coupled with the spontaneous VO formation to preserve charge neutrality, induces downward band bending near the dopant sites. This dynamic aliovalent reconstruction promotes the accumulation of photogenerated holes at the surface, critically enabling the OER [199]. The concept of surface reconstruction must also encompass the dynamic evolution of the cocatalyst semiconductor interface under illumination, since the active phase often diverges from the as-synthesized state due to continuous redox cycling. Abudukade et al. used in situ XAS to reveal the deactivation mechanism of Ni–SrTiO_3_ photocatalysts, first establishing that continuous X-ray exposure itself suppresses the Ni oxidation being measured (likely via X-ray-induced water photolysis generating electrons that quench SrTiO_3_ photoholes), a caution that operando characterization can itself perturb the process it aims to capture [200]. With this artifact minimized, they showed that under UV illumination the as-prepared Ni(0)/Ni(II) core–shell cocatalyst (initially ~4:1 Ni(0):Ni(II)) undergoes progressive hole-driven oxidation, collapsing to a ratio of 0.25 after 10 h, as valence-band holes convert metallic Ni(0) to NiO/Ni(OH)_2_ rather than driving water oxidation, consistent with the substoichiometric H_2_:O_2_ ratio observed [200]. This was confirmed as a purely solid-state restructuring (not dissolution/redeposition) via ICP-MS, XPS, and NiSO_4_ control experiments, and morphologically manifests as flake-like Ni(OH)_2_ growth replacing the smooth core–shell particles. Adding a hole scavenger largely restored stable H_2_ evolution, confirming Ni(0)—not NiO, as some earlier literature proposed as the true active phase [201]. This underscores that active sites are transient and highly sensitive to the local photonic environment, requiring surfaces engineered to either resist photo-oxidation or undergo self-healing reconstruction, analogous to the reversible Rh^3+^/Rh^4+^ couple discussed above [201].

Chemical etching with EDTA enables controlled particle size reduction while maintaining high crystallinity, shortening carrier diffusion lengths and facilitating rapid transport of photogenerated electrons and holes to surface reaction sites. This treatment also produces nano-step surface morphologies that act as catalytically active centers and enhance interactions with adsorbed water molecules [202]. This phenomenon can also be observed for photoelectrochemical water oxidation activity, where nano-cubes of SrTiO_3_ particles in the 10–30 nm range, the water oxidation activity is strongly size-dependent. The specific activity decreases with decreasing particle size and drops exponentially for particles smaller than 20 nm, indicating a trade-off between increased surface area and reduced effectiveness of charge separation or increased defect- related recombination in the smallest crystallites [203]. Similarly, both particle size and crystal shape of Al-doped SrTiO_3_ influence the rate of photocatalytic hydrogen production. In the size range of 250–450 nm, larger crystals generate hydrogen at higher rates (up to ~400 µmol h^−1^ g^−1^) than smaller ones (~200 µmol h^−1^ g^−1^). This is attributed to reduced electron–hole recombination or back-reaction rates in larger particles, where the widths of the space-charge regions become comparable to the particle radius, enabling more effective band bending and charge separation [204].

Dopant engineering plays a crucial role in controlling the photocatalytic performance of SrTiO_3_ by modifying its electronic structure, defect chemistry, and charge-transfer dynamics. The catalytic behavior is strongly dopant-dependent. Al-doped SrTiO_3_ enables overall water splitting (OWS), whereas Rh-doped SrTiO_3_ primarily supports hydrogen evolution. Although both dopants introduce mid-gap states, their functions differ markedly. Al-induced states act as effective hole acceptors, facilitating oxygen evolution by localizing photogenerated holes on surface oxygen species and lowering reaction barriers. In contrast, Rh-induced states remain localized on Rh atoms and do not effectively participate in oxygen evolution [205]. At the bulk level, Al doping introduces hole-trapping states near the valence band that suppress electron–hole recombination without significantly altering the band gap [206], while structural defects such as stacking faults generate internal electric fields that further enhance charge separation and extend carrier lifetimes [207]. Atomistic calculations and spectroscopic studies also show that Al substitution suppresses Ti^3+^ defect states and regulates oxygen vacancy formation, thereby improving carrier migration and surface reaction kinetics [208,209]. Importantly, the positive role of doping is further enhanced by cocatalyst deposition. Rh, Cr_2_O_3_ and CoOOH cocatalysts facilitate rapid electron extraction and favorable energetic realignment at the interface when combined with Al doping [110,210]. Time-resolved spectroscopic analyses confirm that Al doping significantly prolongs carrier lifetimes and reduces recombination centers [211], while operando studies reveal that the combined effects of Al doping and cocatalyst deposition increase the population of long-lived holes and stabilize spatial charge separation. This synergistic defect–cocatalyst engineering is considered a key factor underlying the near-unity apparent quantum yield reported for optimized Al-doped SrTiO_3_ photocatalysts [212].

In addition to Al doping, a wide range of elements have been investigated to tune the photocatalytic properties of SrTiO_3_. Alkali metal doping (Na^+^, K^+^, and Cs^+^) has shown that K-doped SrTiO_3_ exhibits higher Ti^3+^ concentrations and superior intrinsic photocatalytic activity, whereas Na- and Cs-doped systems tend to form deep defect states that act as recombination centers and reduce activity. Density functional theory and synchrotron-based analyses attribute this behavior to differences in A-site ionic polarization, which can localize defect charges and induce uneven structural relaxation, leading to deep-level defects [50,213]. Co-doping strategies have also been explored to optimize defect chemistry [57,214]. For example, La and Al co-doping can reduce detrimental defect states by regulating oxygen vacancies and Ti^3+^ concentrations while promoting lattice stabilization through controlled surface and bulk defect redistribution. Optimized La–Al co-doped SrTiO_3_ loaded with Rh/Cr_2_O_3_/CoOOH cocatalysts has demonstrated enhanced activity and stability for water splitting [57]. Other multi-element doping systems, such as Ir and Sb codoped Al:SrTiO_3_, reveal that photocatalytic performance can be sensitive to operating conditions. Elevated temperatures may accelerate carrier recombination and induce cocatalyst degradation, thereby reducing activity [215]. Overall, these studies highlight that careful control of dopant type, concentration, and defect chemistry is essential for optimizing the photocatalytic performance of SrTiO_3_, and comprehensive reviews on doping strategies have been reported in the literature [106].

Cocatalysts play a crucial role in enhancing the efficiency of SrTiO_3_-based photocatalysts by promoting charge separation and facilitating surface redox reactions. In Rh/CoOOH/SrTiO_3_ systems, Rh is preferentially deposited at the center of crystal facets while CoOOH accumulates near the edges, indicating spatial separation of photogenerated electrons and holes. This intrafacet charge separation significantly improves overall water-splitting activity, leading to a 2.7-fold increase in efficiency compared with SrTiO_3_ containing randomly distributed cocatalysts [216]. In Pt/SrTiO_3_ systems, advanced spectroscopic and microscopic analyses reveal that photogenerated holes preferentially accumulate at the Pt–SrTiO_3_ interface rather than on the oxide surface, identifying this interface as the primary site for water oxidation and highlighting its lower overpotential for the oxygen evolution reaction [217]. However, a single cocatalyst is often insufficient for efficient overall water splitting. For example, the combination of Pt with oxide cocatalysts such as CrO_x_ markedly improves activity by forming a selective layer that suppresses the reverse recombination of H_2_ and O_2_ while facilitating proton transport [218]. Similar stabilization effects are observed in Ni/NiO-based systems, where CrO_x_ deposition enhances catalyst stability and suppresses Ni dissolution during photocatalysis [219]. To reduce reliance on precious metals, alternative cocatalyst systems have also been explored. Pt–Ni alloy nanoparticles combined with CrO_x_ and CoOOH on K-doped SrTiO_3_ have demonstrated photocatalytic performance exceeding that of Rh-based systems due to improved charge transfer through interfacial K–O–Pt bridges [220]. In addition, noble-metal-free cocatalysts such as CoP have shown promising activity by accelerating charge separation and lowering the overpotential for hydrogen evolution. Further deposition of Cr_2_O_3_ effectively suppresses reverse reactions and enhances overall water-splitting efficiency [221].

In addition to compositional modifications, geometric and structural engineering strategies have also been explored to enhance the photocatalytic performance of SrTiO_3_. For instance, hollow SrTiO_3_ architectures with spatially separated cocatalysts have been designed to segregate hydrogen and oxygen evolution sites on the outer and inner surfaces, respectively. Such spatially distributed Pt/SrTiO_3_/CoO_x_ systems promote efficient charge separation and exhibit approximately threefold higher overall water-splitting activity compared with conventional Pt/SrTiO_3_ catalysts [222]. Hierarchical hollow multi-shelled structures have also been developed using La- and Rh-co-doped SrTiO_3_, which extend light absorption to ~520 nm while improving light harvesting and charge separation due to their thin shells and hierarchical morphology. When integrated into a Z-scheme system with BiVO_4_ nanosheets and Pt cocatalysts, these structures demonstrate enhanced solar-to-hydrogen conversion efficiency [223]. Furthermore, one-dimensional SrTiO_3_ nanofibers produced by electrospinning and controlled annealing have shown improved photocatalytic hydrogen evolution. Their porous nanofibrous architecture and optimized oxygen vacancy concentration increase the surface area and suppress charge recombination, thereby providing abundant active sites and facilitating efficient charge transport [224].

In addition to single-component photocatalysts, SrTiO_3_ has been widely integrated into heterojunction and Z-scheme systems to enhance photocatalytic performance through improved charge separation and extended light absorption [225,226,227]. For example, a three-dimensional Au/SrTiO_3_/TiO_2_ hollow nanosphere photocatalyst has been developed through in situ growth of cubic SrTiO_3_ on anatase TiO_2_ hollow structures followed by decoration with plasmonic Au nanoparticles. The synergistic interaction between the plasmon-induced electric field of Au, the internal electric field at the SrTiO_3_/TiO_2_ interface, and the three-dimensional electron transport pathways significantly enhances interfacial charge separation and enables efficient photocatalytic H_2_ evolution without the need for Pt cocatalysts [228]. Similarly, g-C_3_N_4_/SrTiO_3_ type-II heterojunctions decorated with metallic Bi have demonstrated substantially enhanced hydrogen evolution under simulated solar irradiation, where photoreduced Bi^0^ acts as an effective non-noble metal cocatalyst, leading to markedly improved activity and good cycling stability [229]. Other approaches include the use of Ti_3_C_2_ MXene-derived templates to construct TiO_2_/SrTiO_3_ heterostructures, which exhibit efficient charge separation and favorable kinetics for overall water splitting. These studies highlight the potential of heterojunction engineering to further improve the photocatalytic efficiency of SrTiO_3_-based systems [230].

So far, the modification strategies responsible for the exceptional performance of SrTiO_3_-based photocatalysts, particularly CoOOH–Rh/Cr_2_O_3_@SrTiO_3_:Al, have been presented. In principle, these approaches could also be applied to other perovskite photocatalysts. Most reported studies as presented in the following sections have used either pristine perovskites without any modifications or materials modified only with cocatalysts, while low-level Al doping combined with the same cocatalyst engineering strategy has rarely been explored. Only a few studies have adopted parts of this approach. For example, Zhu et al. [231] reported Al-doped SrTaO_2_N, but without employing the same cocatalyst system, whereas other studies on PbTiO_3_ [232,233] applied similar cocatalyst deposition methods without the corresponding doping strategy. These studies demonstrate the feasibility of transferring individual modification strategies to other perovskites; however, the complete modification protocol that proved successful for SrTiO_3_ or even similar ones has not yet been tested for other perovskite families. Therefore, applying this strategy should currently be regarded as a potential research direction rather than an established conclusion.

### 4.2. PbTiO_3_

The photocatalytic behavior of lead titanate (PbTiO_3_) has recently attracted increasing attention, as it lies at the intersection of photocatalysis, ferroelectric materials, and nanotechnology [234]. PbTiO_3_ is a perovskite-type oxide widely known for its ferroelectric and piezoelectric properties; however, it has also emerged as a promising photocatalyst for water splitting. Compared with SrTiO_3_, PbTiO_3_ possesses a smaller band gap, which enhances its ability to harvest visible light while maintaining conduction and valence band positions suitable for both HER and OER reactions.

**Figure 3:**
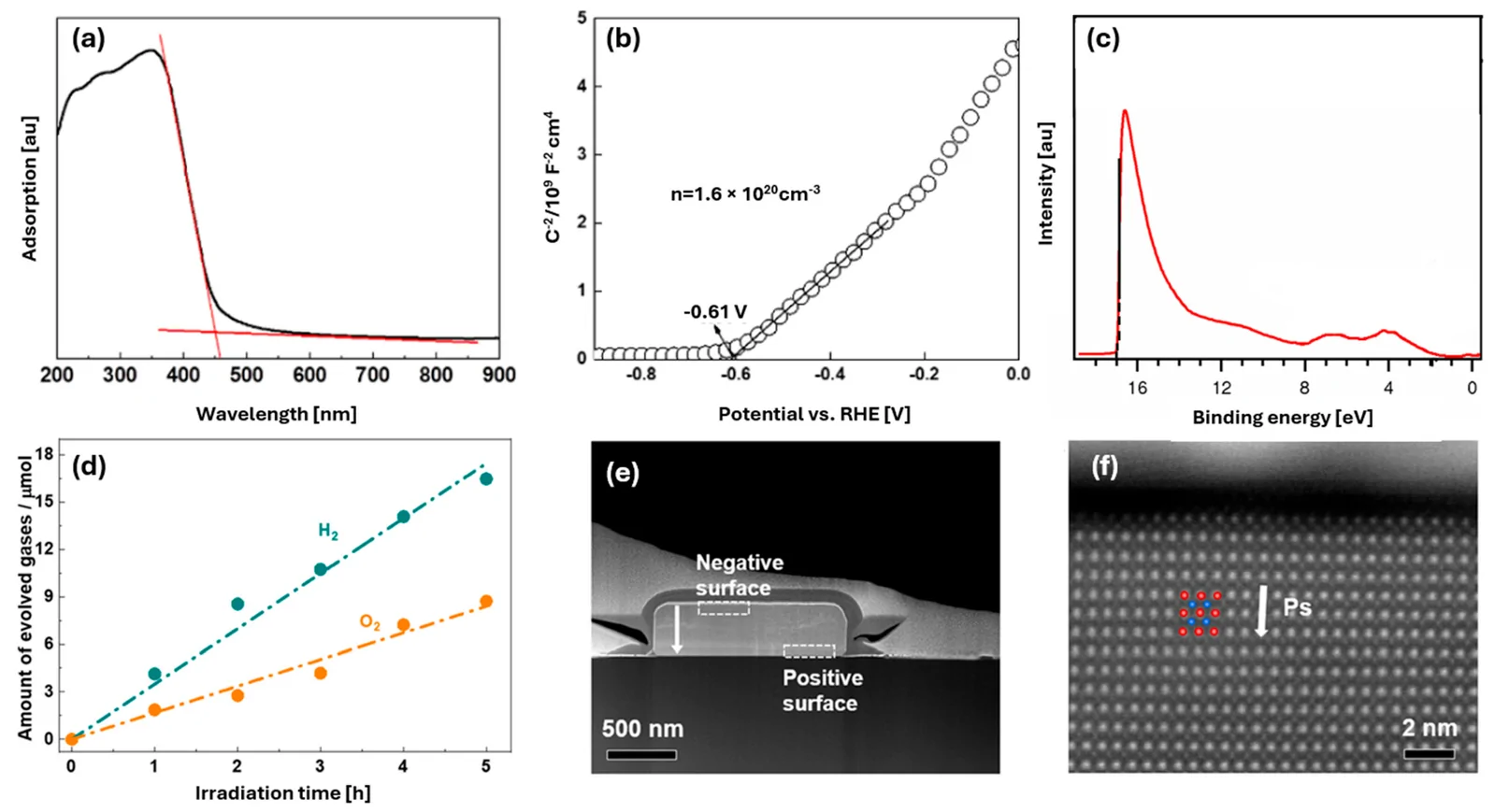
(**a**) UV–vis absorption spectrum of PbTiO_3_; (**b**) Mott–Schottky plot of PbTiO_3_, (**c**) UPS spectrum of PbTiO_3_; (**d**) photocatalytic overall water-splitting performance of PbTiO_3_/Rh/Cr_2_O_3_ under simulated solar irradiation; (**e**) cross-sectional view of PbTiO_3_ prepared by focused ion beam (FIB) cutting; (**f**) atomic-resolution cross-sectional HAADF-STEM image of PbTiO_3_, where red spheres denote Pb atoms, blue spheres represent Ti atoms, and the arrow indicates the polarization direction, Reprinted with permission from [235,238]. Copyright 2022 American Chemical Society.

Optical characterization (Figure 3) shows that pristine PbTiO_3_ begins absorbing light at approximately 460 nm, corresponding to a band gap of about 2.7 eV. In contrast, SrTiO_3_ absorbs primarily below ~370 nm. Consequently, PbTiO_3_ can utilize a portion of the visible spectrum, enabling significantly greater solar-energy harvesting. In practical terms, SrTiO_3_ is capable of absorbing less than approximately 5% of the solar spectrum, whereas PbTiO_3_ can theoretically absorb more than 10%. This enhanced light absorption increases the probability of photogenerated electron–hole pair formation and improves the potential for effective charge separation. Electrochemical characterization using Mott–Schottky analysis indicates that PbTiO_3_ behaves as an n-type semiconductor with a flat-band potential of approximately −0.6 V vs. RHE. This corresponds to a conduction band minimum near −0.4 V vs. RHE and a valence band maximum around +2.3 V vs. RHE. As a result, both the hydrogen evolution potential (0 V vs. RHE) and the oxygen evolution potential (1.23 V vs. RHE) lie within the band gap, indicating that PbTiO_3_ possesses sufficient thermodynamic driving force for overall water splitting [235]. Ultraviolet photoelectron spectroscopy measurements further reveal a work function of approximately 3.7 eV for PbTiO_3_, which is slightly lower than that of SrTiO_3_ (~4 eV), suggesting that photoexcited electrons will be emitted more readily [236]. Additionally experimental studies have confirmed the capability of PbTiO_3_ to drive photocatalytic water splitting under both ultraviolet and visible irradiation. Wan et al. [235]. demonstrated overall water splitting in pure water without the use of sacrificial agents by loading Rh/Cr_2_O_3_ as a reduction cocatalyst onto PbTiO_3_. Under irradiation from a 300 W xenon lamp, hydrogen and oxygen evolution rates of 33 and 17 μmol g^−1^ h^−1^, respectively, were obtained. In another study [233], overall water splitting was achieved under visible-light irradiation using a 300 W xenon lamp equipped with a 420 nm cutoff filter. In this system, 0.001 M FeCl_3_ was dissolved in water and a mixed photocatalyst consisting of RuO_2_/Na,Mo-doped PbTiO_3_ and RhCrO_x_/PbTiO_3_ was employed, resulting in hydrogen and oxygen evolution rates of 36 and 18 μmol g^−1^ h^−1^, respectively. In addition to overall water splitting, the photocatalytic activity of PbTiO_3_ has also been investigated for half-reactions. Under visible-light irradiation, Pt-loaded PbTiO_3_ using methanol as a sacrificial electron donor produced hydrogen at a rate of 27.4 μmol g^−1^ h^−1^, while RuO_2_-loaded PbTiO_3_ in the presence of AgNO_3_ as a sacrificial electron acceptor yielded oxygen at a rate of 183 μmol g^−1^ h^−1^ [237]. These studies collectively highlight the potential of PbTiO_3_ as a promising photocatalyst for overall photocatalytic water splitting.

Numerous studies have been conducted to elucidate the mechanisms underlying the photocatalytic activity and charge-carrier (electron–hole) separation [239,240,241]. PbTiO_3_ is a ferroelectric perovskite that possesses intrinsic internal electric fields due to spontaneous polarization in its ferroelectric phase, which arises from the off-center displacement of Ti^4+^ within the TiO_6_ octahedra and the relative shift in Pb^2+^ ions along the crystallographic c-axis when the structure becomes tetragonal below the Curie temperature. These ionic displacements generate a permanent dipole moment in each unit cell, leading to very strong microscopic (local) internal electric fields that stabilize the ferroelectric distortion; however, in an ideal bulk crystal at equilibrium, these do not result in a net macroscopic internal electric field because the bound surface charges associated with polarization are compensated by ferroelectric domain formation, free charge screening, or surface and interface reconstruction. The polarization direction defines the electrical nature of the crystal facets. Only the surfaces perpendicular to the c-axis are intrinsically polar, with the (001) surface being positively polarized (P^+^) and the (00-1) surface being negatively polarized (P^−^) for a given polarization orientation, while side facets such as (100), (010), and (110) are non-polar because the polarization lies parallel to them; importantly, the sign of the surface polarization can be reversed by switching the ferroelectric polarization direction. When PbTiO_3_ is irradiated with light of energy equal to or greater than its band gap, electron–hole pairs are generated, and the intrinsic polarization field immediately separates these carriers without any external bias, driving electrons toward the P^+^ surface and holes toward the P^−^ surface, thereby strongly suppressing recombination. This polarization-driven charge separation under illumination gives rise to the ferroelectric (bulk) photovoltaic effect, which can produce photovoltages exceeding the band gap and leads to pronounced facet-dependent photoactivity. These effects are further enhanced in thin films, single-domain crystals, poorly screened surfaces, and at domain walls, where local electric fields are especially strong, making ferroelectric PbTiO_3_ a uniquely powerful material for light-driven charge separation, surface chemistry, and photo-catalytic applications [242,243,244,245]. With the help of spatially resolved surface photovoltage spectroscopy, it is clearly demonstrated that the depolarization field in single-domain ferroelectric PbTiO_3_ nanoplate is the main driving force for charge separation and it can effectively drive photogenerated electrons and holes to the positive and negative polarization facets, respectively. Moreover, the charge separation ability of PbTiO_3_ nanoplates increases with increasing particle size along the polarization direction, due to the increasing potential difference between the opposite polarization facets. Consequently, this driving force for charge separation directly contributes to the enhancement of the photocatalytic hydrogen evolution reaction activity in ferroelectrics [239]. More recently, in ferroelectric SrTiO_3_/PbTiO_3_ nanoplate heterostructures, increasing the average thickness of the single-domain PbTiO_3_ nanoplates from 30 to 107 nm enhanced the overall water-splitting performance by up to 22 times. The larger thickness increases the spontaneous polarization strength, resulting in a stronger incompletely compensated depolarization field that more effectively drives the separation of photogenerated carriers. Concurrently, the concentration of oxygen vacancies (which promote the oxygen evolution reaction) also increases with thickness [246].

**Figure 4:**
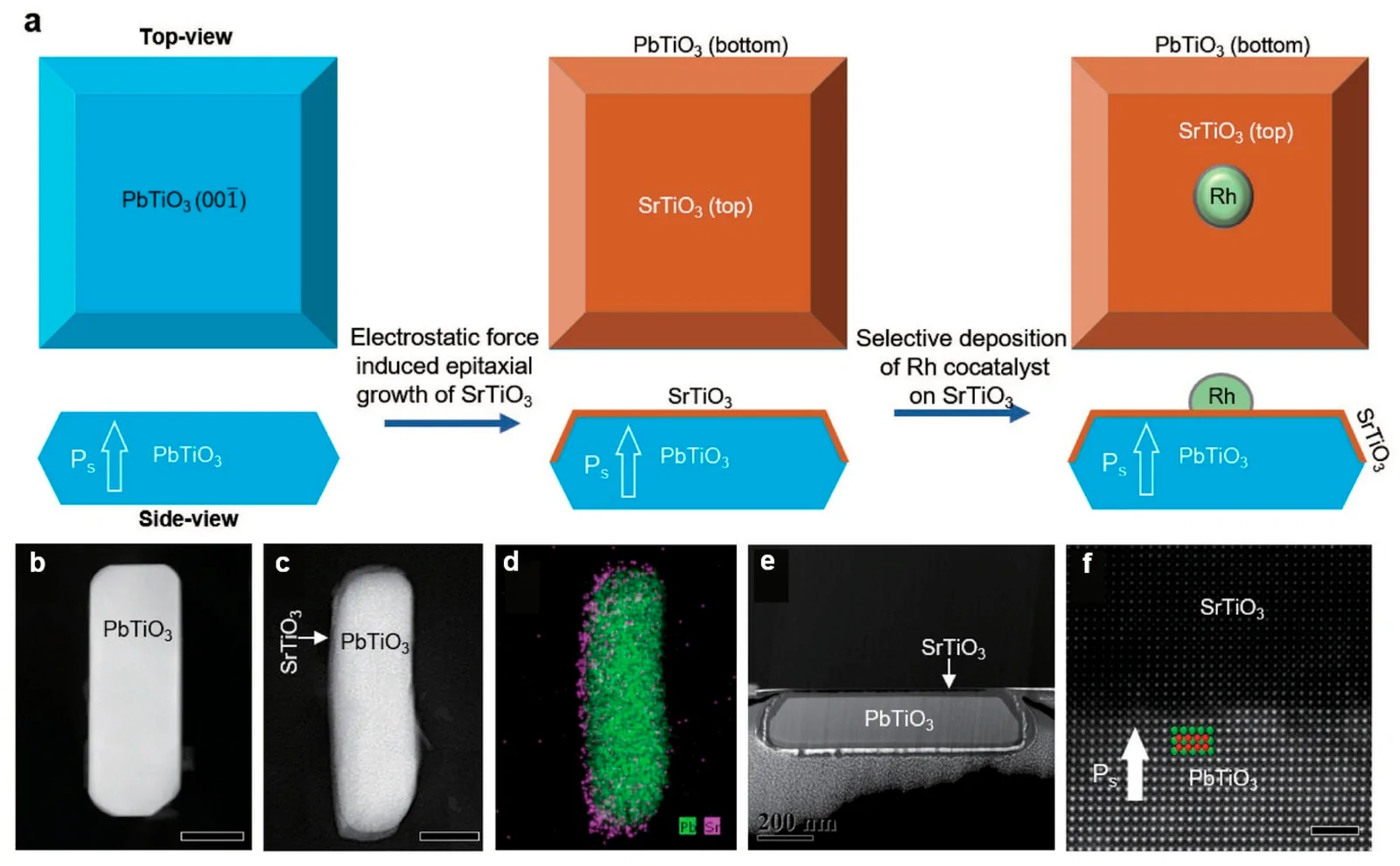
Structural characterization of the PbTiO_3_/SrTiO_3_ heteroepitaxial architecture. (**a**) Schematic illustration of the heteroepitaxial growth process governed by polarization screening. (**b**–**f**) Cross-sectional HAADF-STEM images of the PbTiO_3_/SrTiO_3_ heterostructure; green spheres represent Pb atoms, red spheres denote Ti atoms, and the arrow indicates the polarization direction [247].

In addition to the internal polarization field that promotes charge separation in PbTiO_3_, surface defects, particularly oxygen vacancies, play a crucial role in determining photocatalytic activity. The concentration of oxygen vacancies can be tuned through thermal annealing, and both experimental and theoretical studies indicate that these defects act as active sites for water oxidation to O_2_, while vacancy-free surface sites tend to favor H_2_O_2_ formation. The magnitude of ferroelectric polarization also strongly influences the photocatalytic performance [235]. To further exploit these effects, heteroepitaxial SrTiO_3_ layers have been grown on polarized PbTiO_3_ nanoplates (Figure 4). Driven by electrostatic interactions with the polarized surfaces, this structure promotes a redistribution of defects, enriching Ti^3+^ species near the positively polarized surface and oxygen vacancies near the negatively polarized surface. In the resulting PbTiO_3_/SrTiO_3_ heterostructure, the vacancy-rich negatively polarized surface acts as an efficient water oxidation site, while the positively polarized surface facilitates cocatalyst dispersion and lowers the Schottky barrier, enabling more efficient charge extraction. Consequently, the optimized heteroepitaxial structure exhibits significantly enhanced and stable overall water-splitting activity, reaching approximately fifteen times higher performance than pristine PbTiO_3_ nanoplates [247]. Furthermore, the growth of SrTiO_3_ nanolayers on polarized facets has been shown to suppress interfacial Ti defects and establish efficient electron-transfer pathways, extending electron lifetimes from tens of microseconds to the millisecond range and thereby increasing the participation of photogenerated electrons in water-splitting reactions [232]. The role of surface oxygen vacancies in water splitting on PbTiO_3_ has also been investigated through theoretical studies. Calculations indicate that both the concentration and spatial arrangement of oxygen vacancies are critical for water-splitting reactions on the PbTiO_3_ (001) surface. Neighboring oxygen vacancies can act as active sites that stabilize adsorbed water molecules and facilitate O–H bond dissociation. For TiO_2_-terminated surfaces, multiple adjacent vacancies are required to enable water dissociation, but excessive vacancy formation can trap hydrogen atoms and hinder H_2_ evolution. In contrast, PbO-terminated PbTiO_3_ surfaces more readily support hydrogen formation due to weaker Pb–O bonding, although the generated H_2_ molecules remain weakly bound near the surface. These studies suggest that both entropic constraints, stemming from the requirement of clustered vacancies, and enthalpic barriers associated with vacancy formation or H_2_ desorption can limit overall reaction efficiency [248]. Complementary first-principles calculations and ab initio molecular dynamics simulations further show that water preferentially undergoes dissociative adsorption on the PbTiO_3_ (001) surface, and that the presence of oxygen vacancies significantly enhances this process. Vacancies stabilize hydroxyl intermediates and increase surface OH coverage, thereby improving the reactivity of defective PbTiO_3_ surfaces for water-splitting reactions [249].

In addition to pristine PbTiO_3_, several modification strategies, including elemental doping, have been investigated to enhance its photocatalytic performance. For example, Ta-doped PbTiO_3_ exhibits improved charge separation and significantly enhanced photocatalytic overall water-splitting activity when combined with suitable cocatalysts [250]. Furthermore, PbTiO_3_ has been widely integrated with other materials to form Z-scheme systems and heterojunction structures. A summary of the materials reported in combination with PbTiO_3_ is presented in Table 2. As an illustrative example, the selective growth of TiO_2_ nano-islands on specific facets of PbTiO_3_ has been shown to dramatically enhance photocatalytic performance, increasing hydrogen production rates by more than an order of magnitude. This improvement is primarily attributed to improved interfacial charge separation and more efficient utilization of photogenerated carriers [251,252].

From an environmental perspective, despite its promising photocatalytic properties, the practical application of PbTiO_3_ raises concerns due to the presence of lead. Although PbTiO_3_ generally exhibits good chemical stability during photocatalytic water splitting [241], the potential release of Pb^2+^ ions under long-term operation has not been systematically investigated. Therefore, future studies should evaluate Pb leaching and develop strategies to minimize any potential environmental impact while maintaining high photocatalytic performance.

Overall, as evident from Table 2 and the literature reviewed, the modification strategy that has proven highly effective for Al-doped SrTiO_3_ has not yet been extended to PbTiO_3_. Studies considering both doping and appropriate cocatalyst deposition remain very limited. As summarized in Table 2, only one study investigated the doping effect using Na and Mo [233]. However, a total doping level of 15% was employed, which is considerably higher than the approximately 1% doping level used by Takata et al. Analogous to silicon solar cells, where excessive dopant concentrations can alter the internal electric field of the crystal, such a high doping level in PbTiO_3_ may substantially modify the parent material and its intrinsic crystal and electronic structure. Therefore, this highly doped system cannot be directly compared with the low-level doping strategy applied to SrTiO_3_. Moreover, other dopants, particularly in combination with optimized cocatalyst deposition, have not yet been systematically investigated for PbTiO_3_. This represents a clear gap in the current literature. Considering that PbTiO_3_ possesses a narrower band gap than SrTiO_3_, exhibits intrinsic spontaneous polarization that can promote charge separation, and has suitable band-edge positions for overall water splitting, it can be a candidate for benefiting from the same modification strategy that has proven effective for SrTiO_3_. Therefore, investigating the doped, cocatalyst-modified PbTiO_3_ further is both scientifically justified and potentially valuable for developing more efficient visible-light-driven photocatalysts.

## 5. Double Perovskites

Double perovskites constitute an important subclass of perovskite materials with the general chemical formula A_2_BB′X_6_, where A is typically a monovalent or divalent cation (commonly an alkali or alkaline-earth metal), B and B′ are two different metal cations occupying alternating positions in the octahedral framework, and X is usually an oxide or halide anion [264]. Structurally, double perovskites derive from the classical ABX_3_ perovskite lattice, but with an ordered arrangement of two distinct B-site cations in a rock-salt configuration. This ordering doubles the unit cell and introduces additional structural and electronic degrees of freedom that allow fine-tuning of the material’s electronic, optical, and catalytic properties. The crystal structure of double perovskites consists of a three-dimensional framework of corner-sharing BX_6_ and B′X_6_ octahedra. The A-site cations occupy the cuboctahedral cavities formed by the octahedral network and primarily stabilize the structure through ionic interactions [265]. Structural stability is commonly described using the extended Goldschmidt tolerance factor, which depends on the ionic radii of the A, B, B′, and X ions. Differences in ionic size, charge, and electronegativity between the B and B′ cations can induce structural distortions such as octahedral tilting or lattice strain, which in turn strongly influence the electronic band structure and charge transport properties [266].

One of the major advantages of double perovskites is the tunability of their electronic structure. In many systems, the valence band is primarily derived from hybridized anion p orbitals and the d orbitals of one B-site cation, while the conduction band mainly originates from the d orbitals of the second B′ cation. Because the B and B′ cations typically differ in oxidation state and electronic configuration, their orbitals contribute asymmetrically to the band edges [267]. This orbital differentiation is beneficial for photocatalysis, as it promotes spatial separation of photogenerated charge carriers. Upon light absorption, electrons are excited to the conduction band associated with the B′ cation, while holes remain localized near the B-centered octahedra. Such intrinsic separation suppresses electron–hole recombination and extends carrier lifetimes. In addition, the ordered B/B′ arrangement can create internal electric fields or asymmetric charge distributions that further facilitate directional charge transport. In some cases, double perovskites may also exhibit type-II–like band alignment between different atomic sublattices, further enhancing charge separation efficiency [268].

Ability to independently vary the A-site cation, the B/B′ cation combination, and the anion composition enables precise control over key material properties such as band gap, carrier mobility, defect tolerance, and chemical stability. This compositional flexibility has made some of the double perovskites suitable for photocatalytic water splitting. For instance, La_2_FeTiO_6_ and Sr_2_CoTaO_6_ have been reported to act as photoactive catalysts for water splitting in the presence of sacrificial agents, even without the addition of cocatalysts [269,270]. As an illustration, Sr_2_CoTaO_6_ behaves as an n-type semiconductor with a band gap of approximately 2.7 eV, while the conduction band minimum and valence band maximum are located at −0.87 V and +1.83 V versus the normal hydrogen electrode (NHE, pH = 7), respectively. These band positions provide sufficient thermodynamic driving force for photocatalytic water splitting. Nevertheless, the introduction of cocatalysts such as RuO_2_ or Rh can significantly enhance the photocatalytic activity by improving charge separation and accelerating surface redox reactions [271]. Beyond cocatalyst loading, cation doping represents an effective strategy for improving the photocatalytic performance of double perovskites. For example, tungsten doping in Sr_2_FeNbO_6_ has been reported to increase hydrogen production by nearly threefold. A relatively small substitution level, such as Sr_2_FeNb_0.99_W_0.01_O_6_, can substantially enhance activity and selectivity while preserving the structural integrity of the perovskite lattice. Importantly, this low doping concentration does not significantly distort the crystal structure, allowing the material to retain its favorable electronic and structural characteristics [272].

In addition to composition and doping, the synthesis method can strongly influence the photocatalytic performance of double perovskites. For example, studies on Sr_2_FeTaO_6_ have shown that high-temperature synthesis can introduce defects associated with high oxidation-state Fe species, which negatively affect photocatalytic activity [269]. However, a simple post-synthetic thermal treatment in the presence of ethylene glycol can effectively remove these defect states and improve catalytic performance [273]. Similarly, the preparation route has been shown to influence the activity of Sr_2_CoTaO_6_. A sample synthesized via a low-temperature flux method exhibited bifunctional photocatalytic activity for both the HER and OER reactions under visible-light irradiation, even in the absence of cocatalysts. In contrast, samples prepared through conventional high-temperature solid-state synthesis showed significantly lower photocatalytic activity [271]. Double perovskites also benefit from morphology-dependent photocatalytic behaviour through the synergistic effects of cation ordering and surface structure. Kumar et al. demonstrated that CaLaScRuO_6+_δ functions as an efficient and stable photocatalyst for the oxygen evolution reaction under visible-light irradiation (λ = 420 nm) at neutral pH. FESEM analysis revealed agglomerated polycrystalline particles with a broad size distribution, providing abundant accessible surface area for water adsorption while exposing mixed-valence Ru active sites. Together with B-site disorder, the particle morphology and structural homogeneity promoted efficient charge transfer and facilitated visible-light-driven oxygen evolution [274].

To conclude, double perovskites offer greater compositional flexibility than simple ABO_3_ perovskites because two different cations occupy the B-site, allowing independent tuning of the electronic structure, band gap, and band-edge positions. This structural versatility has enabled the development of several visible-light-responsive photocatalysts with improved charge separation through ordered B-site cation arrangements. Furthermore, the large compositional space provides opportunities to tailor defect chemistry and optimize catalytic active sites. However, despite these advantages, most reported double perovskites still exhibit photocatalytic activities that are insufficient for efficient overall water splitting. Many require sacrificial reagents and only demonstrate hydrogen or oxygen evolution half-reactions [271,271,275]. In addition, achieving a high degree of B-site ordering is synthetically challenging, and cation disorder can introduce recombination centers that reduce charge-carrier lifetimes. Table 3 summarizes the majority of double perovskites so far have been reported for photocatalytic water-splitting applications. As obvious their photocatalytic activities have predominantly been evaluated only in the presence of sacrificial agents and mainly for half-reactions such as HER or OER. This suggests that many of these materials may not be intrinsically active for overall water splitting.

## 6. Oxynitride Perovskites

Oxynitride perovskites have emerged as an important class of materials for visible-light-driven photocatalysis, particularly in applications such as photocatalytic water splitting and solar fuel production [280]. These materials generally adopt a perovskite-derived crystal structure with the formula ABO_2_N or ABON_2_, where A is typically an alkaline-earth or rare-earth cation (e.g., La, Ba, Sr), B is a transition metal cation (e.g., Ta, Nb, Ti), and the anion lattice contains both oxygen and nitrogen. Structurally, they consist of a three-dimensional network of corner-sharing BO_6−x_N_x_ octahedra, with the A-site cations occupying the larger cuboctahedral cavities of the lattice. The incorporation of nitrogen into the oxide lattice plays a key role in determining the electronic structure of these materials [281]. Perovskite oxides generally possess wide band gaps that restrict light absorption to the ultraviolet region. Typical examples include SrTiO_3_ with a band gap of approximately 3.2 eV (absorption edge < 380 nm) and NaTaO_3_ with a band gap of approximately 4.1 eV [282]. In these materials, the valence band is primarily composed of O 2p orbitals, which lie relatively low in energy. In contrast, the incorporation of nitrogen into perovskite oxynitrides introduces N 2p orbitals that hybridize with O 2p orbitals, raising the valence-band maximum while leaving the conduction-band minimum largely unchanged, thereby narrowing the band gap. Consequently, perovskite oxynitrides typically exhibit band gaps in the range of 1.8–2.5 eV and absorb a much larger portion of the visible spectrum [283]. Representative absorption edges include approximately 560 nm for SrTaO_2_N, 620 nm for LaTiO_2_N (Eg ≈ 2.1 eV), 660 nm for BaTaO_2_N, 680 nm for SrNbO_2_N, and up to 740 nm for BaNbO_2_N [281,284]. This band-gap reduction enables oxynitride perovskites to harvest a substantially larger fraction of solar energy than oxide perovskites, making them more promising candidates for visible-light-driven overall water splitting. Furthermore, the band-gap energy decreases with increasing effective electronegativity of the B-site cation (Nb > Ta) and decreasing A-site cation size [282]. Importantly, despite their narrower band gaps, the band-edge positions of most perovskite oxynitrides still straddle the water redox potentials, satisfying the thermodynamic requirements for overall water splitting under visible-light irradiation. However, this enhanced visible-light absorption is accompanied by trade-offs. The upward shift in the valence band reduces the oxidation driving force, while the incorporation of nitrogen can introduce defect states that promote charge-carrier recombination. Therefore, although oxynitride perovskites offer advantages over oxide perovskites in solar-light utilization, achieving high photocatalytic efficiencies still requires careful control of defects, crystallinity, and cocatalyst engineering.

From the stability point, perovskite oxides exhibit excellent chemical stability and strong resistance to photocorrosion. For example, Al-doped SrTiO_3_ has been reported to sustain overall water splitting for more than 1000 h under continuous illumination [285]. Perovskite oxynitrides are typically synthesized at high temperatures under flowing ammonia, which generally results in highly crystalline materials with good structural stability. They also exhibit excellent chemical inertness in aqueous electrolytes over a wide pH range, including strongly alkaline conditions (pH ≥ 13), can withstand aggressive acids such as boiling aqua regia, and remain thermally stable in air up to several hundred degrees Celsius [1,3]. The main stability challenge of oxynitride perovskites is not their chemical stability but their potential susceptibility to photocorrosion. Under illumination, photogenerated holes may oxidize N^3−^ to N_2_, creating nitrogen vacancies that can subsequently be occupied by oxygen atoms, thereby modifying the surface chemistry and photocatalytic activity [285]. However, this behavior is not universal and strongly depends on the oxynitride composition, crystal quality, reaction conditions, and cocatalyst. While some oxynitrides exhibit continuous nitrogen loss and gradual deactivation [286], others release only a small amount of N_2_ during the initial stages before reaching a stable state [287], and some show negligible nitrogen evolution under operating conditions [288,289]. Therefore, the evolution of N_2_ should be routinely monitored when evaluating the long-term stability of oxynitride photocatalysts, rather than assuming that all oxynitrides undergo severe photocorrosion. Also some materials such as LaTiO_2_N, SrTaO_2_N, and BaTaO_2_N employ cocatalysts (e.g., CoO_x_) to suppress self-oxidation and further improve stability [282,285].

**Figure 5:**
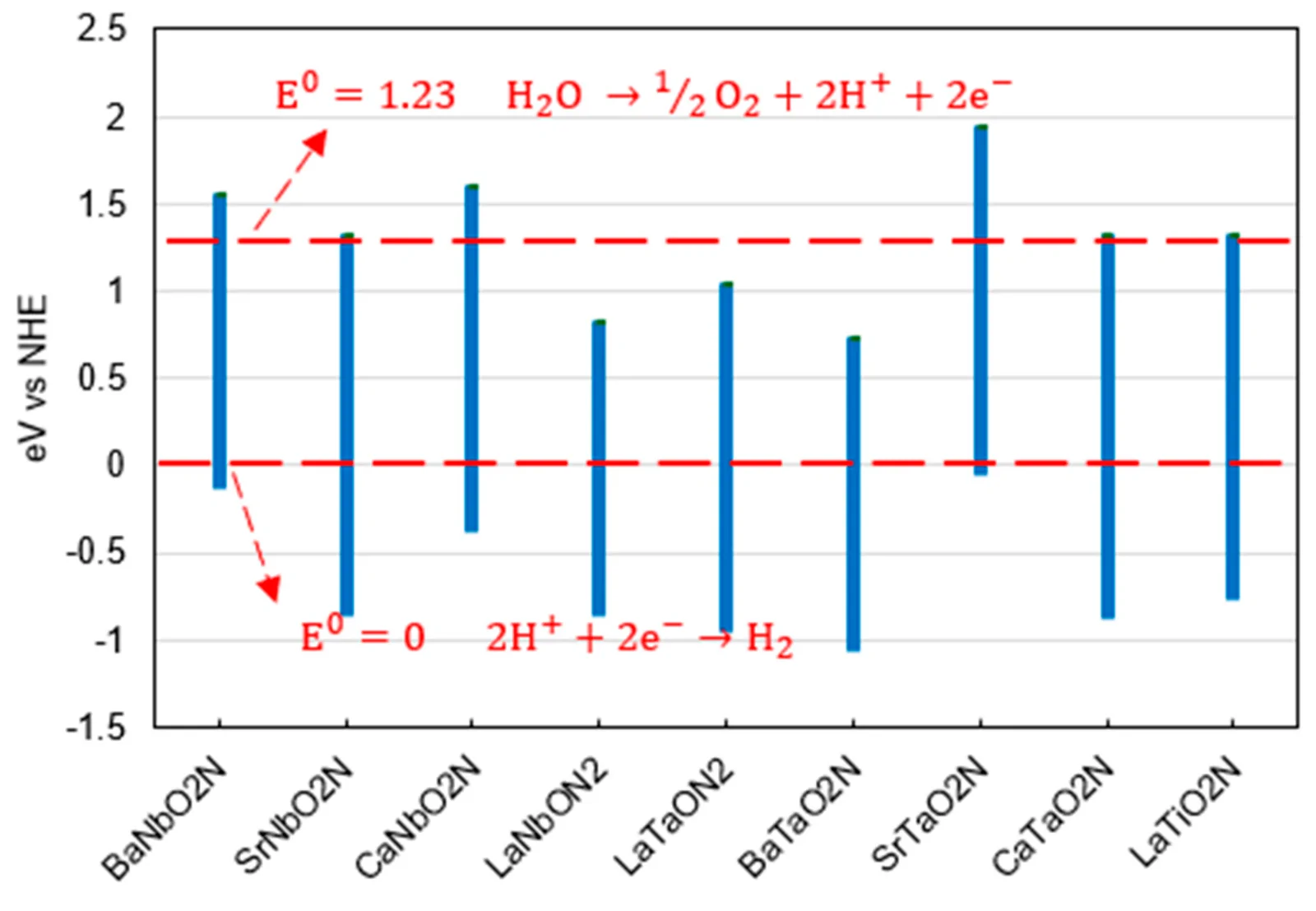
Conduction and valence band edge positions of oxynitride perovskite relative to the normal hydrogen electrode (NHE) [62,288,297,298,299,300,301].

Oxynitride perovskites have been widely investigated as photocatalysts [280,290,291,292,293]. These materials have demonstrated promising activity for photocatalytic water splitting, where they can drive the HER, OER, or both under visible-light irradiation. Figure 5 illustrates the conduction and valence band positions of various perovskites relative to the water redox potentials. As shown, most oxynitride perovskites exhibit more favorable band edge alignment for both HER and OER reactions compared with their oxide counterparts. This improved band positioning, together with their narrower band gaps, makes oxynitride perovskites more suitable for visible-light-driven photocatalytic water splitting. Concerns regarding possible nitrogen loss during photocatalysis have also been investigated, and experimental studies generally show that these materials maintain structural stability without significant evolution of N_2_ [294,295,296].

The electronic band structure of oxynitrides also contributes to efficient charge separation. The valence band is mainly composed of hybridized N 2p and O 2p orbitals, while the conduction band is dominated by transition-metal d orbitals such as Ta 5d or Ti 3d. This difference in orbital character can reduce electron–hole recombination and facilitate charge transport [283,284]. In addition, lattice distortions and polarization effects may generate internal electric fields that further promote charge separation. Surface facet-dependent charge distribution can also occur, allowing reduction and oxidation reactions to take place at different sites on the photocatalyst surface. The use of cocatalysts is often necessary to enhance charge separation and reaction kinetics. Noble metals such as Pt can act as electron sinks and promote hydrogen evolution, while metal oxides such as RuO_2_ or NiO facilitate oxygen evolution [35,302]. Selective deposition of these cocatalysts improves the extraction of photogenerated carriers and suppresses recombination.

Various strategies have been explored to further improve the performance of oxynitride photocatalysts, including defect engineering, surface modification, nanostructuring, and elemental doping. Among these, Mg doping has been one of the most widely studied approaches [61,303,304]. Mg has been introduced into several oxynitride perovskites, including LaTiO_2_N [304], BaTaO_2_N [294], SrTaO_2_N [303], and CaTaO_2_N [294], often resulting in enhanced photocatalytic activity. In many cases, Mg^2+^ substitutes the B-site transition-metal cation (e.g., Ti^4+^ or Ta^5+^), creating charge imbalance that modifies carrier concentration and suppresses electron–hole recombination. Other dopants such as Ca, Ta, and Nb have also been explored to tailor the electronic structure and defect chemistry of these materials. For example, Ca substitution can influence lattice distortion and local coordination environments, while variations in Ta content can modify the conduction band structure [305,306]. Through careful control of dopant type and concentration, the band structure and carrier dynamics of oxynitride perovskites can be optimized to improve photocatalytic efficiency. In addition to compositional tuning, the reaction conditions used to evaluate photocatalytic performance are important. Many studies use sacrificial reagents, such as methanol for HER or AgNO_3_ for OER, to study individual half-reactions [307,308]. However, these systems do not represent true overall water-splitting conditions. To better mimic practical operation, redox shuttle mediators such as Fe^2+^/Fe^3+^, I^−^/IO_3_^−^, and Fe(CN)_6_^4−^/Fe(CN)_6_^3−^ have been employed, often in combination with buffer solutions such as sodium phosphate [287,304,309].

Efficient overall water splitting is frequently achieved using a two-photocatalytic system, in which two different photocatalysts are used for HER and OER. In many reported systems, SrTiO_3_-based photocatalysts modified with noble metals such as Rh, Ru, or Pt serve as the hydrogen-evolving photocatalyst, while oxynitride perovskites act as the oxygen-evolving component. A representative example is the system combining Mg-doped LaTiO_2_N with Ru/Rh-loaded SrTiO_3_, using the Fe^2+^/Fe^3+^ redox couple as an electron mediator. Under simulated solar irradiation (AM 1.5 G), this configuration achieved overall water splitting with hydrogen and oxygen evolution rates of approximately 60 μmol g^−1^ h^−1^ and 30 μmol g^−1^ h^−1^, respectively, representing one of the highest reported activities for oxynitride-based systems [304]. Furthermore, the synthesis and post-treatment conditions of oxynitride perovskites can strongly influence their photocatalytic properties. For instance, post-treatment of BaTaO_2_N with NH_3_ at elevated temperatures has been shown to enhance photocatalytic activity by improving nitrogen incorporation, crystallinity, and anion ordering while reducing surface defects. These structural improvements reduce recombination centers and facilitate more efficient charge transport to catalytic sites [310].

For perovskite-type oxynitride BaNbO_2_N, reducing the particle size (derived from starting oxide particles ranging from ~1.05 to 6.92 μm) significantly increases the specific surface area and shortens the diffusion distance of photogenerated charges. This enhances the efficiencies of charge generation, separation, and collection, leading to higher photocurrent densities; the smallest particles produced the highest photocurrent of 2.20 mA cm^−2^ at 1.23 V_RHE_ under simulated sunlight [311]. Evidence from oxynitride perovskites further highlights the importance of crystallographic orientation in determining photocatalytic activity. In LaTiO_x_N_γ_ thin-film model systems, crystallographic surface orientation strongly influences photocatalytic water-splitting performance after surface stabilization: the absorbed photon-to-current conversion efficiency (APCE) of epitaxial films differs by ~50% between orientations and reaches values up to five times higher than polycrystalline samples, with the (001) surface delivering the highest APCE (nearly three times that of the predominant (112) orientation). This enhancement arises because higher crystalline quality reduces bulk recombination centres, while the (001) facet preferentially exposes a low-energy LaN termination that is more resistant to oxidation and places nitrogen-derived valence-band states at higher energy. The resulting favourable energetic landscape promotes downhill migration of photogenerated holes to the solid–liquid interface, thereby accelerating the oxygen-evolution reaction relative to mixed-cation (112) surfaces that contain a lower accessible N:O ratio [312].

Compared with oxide perovskites, oxynitrides offer a significant advantage by extending light absorption into the visible region through nitrogen incorporation, making them considerably more suitable for solar-driven photocatalysis. Several oxynitride perovskites, including LaTiO_2_N, LaTaON_2_, BaTaO_2_N, and CaTaO_2_N, have proven to satisfy the fundamental thermodynamic requirements for overall water splitting [61,294,313,314]. However, this improved optical performance may be accompanied by increased synthetic complexity and reduced stability, although the extent of this limitation is strongly material dependent. Therefore, the stability of each material should be carefully evaluated under photocatalytic operating conditions. Table 4 shows the photocatalytic activity of oxynitride perovskites.

## 7. Ruddlesden–Popper (RP) Perovskites

Ruddlesden–Popper (RP) perovskites constitute an important family of layered oxide materials that have attracted considerable attention in photocatalysis, particularly for photocatalytic water splitting. These compounds follow the general formula A_n+1_B_n_O_3n+1_, where A is typically an alkali or alkaline-earth metal (e.g., Na, K, Ca, Sr, Ba), B is a transition metal such as Ti, Nb, or Ta, and n denotes the number of perovskite layers in the structure. Structurally, RP phases consist of perovskite-like slabs (ABO_3_) composed of corner-sharing BO_6_ octahedra, separated by rock-salt AO layers along the crystallographic c-axis. This layered architecture introduces structural anisotropy and forms interlayer regions that can facilitate ion transport and provide accessible sites for surface reactions [326,327].

The valence band in RP-type perovskites is primarily composed of O 2p orbitals, whereas the conduction band is mainly derived from the d orbitals of transition metals such as Nb 4d or Ta 5d. Several layered tantalates and niobates, including KCa_2_Nb_3_O_10_, NaCa_2_Ta_3_O_10_, and Sr_2_Ta_2_O_7_, exhibit band gaps in the range of approximately 3.0 and 3.8 eV, making them mainly active under ultraviolet irradiation [328,329,330]. Despite their relatively wide band gaps, these materials often demonstrate photocatalytic activity due to their charge transport properties and high chemical stability. Upon photoexcitation, electrons are promoted from the valence band to the conduction band, generating electron–hole pairs. The layered structure facilitates charge separation through anisotropic charge transport within the perovskite slabs and spatial separation across the interlayer regions. In addition, structural distortions and differences between the perovskite blocks and rock-salt layers can generate internal electric fields that further suppress electron–hole recombination [47,331,332,333].

In photocatalytic water splitting, RP perovskites are most employed and reported for hydrogen evolution reactions under UV irradiation, typically in the presence of cocatalysts such as Pt, NiO, or RuO_2_, which enhance surface reaction kinetics and facilitate proton reduction [334,335,336]. Various strategies have been explored to improve their photocatalytic performance, among which elemental doping is one of the most widely applied approaches. For example, Ga doping in K_2_LaTa_2_O_6_N has been reported to significantly enhance photocatalytic activity. In Ga-doped K_2_LaTa_2_O_6_N, a trade-off between Ga concentration and photocatalytic performance was observed. Ga incorporation reduced particle size and suppressed charge recombination, but excessive doping led to band gap widening, which is unfavorable for visible-light absorption [337]. Other dopants, including Pd, Cr, and Rh, have also been investigated to modify the electronic structure and improve photocatalytic efficiency [334,338]. In addition, co-doping strategies, such as La/Rh [339] and La/Fe [340] substitution, have been reported to further enhance photocatalytic activity for water splitting. The synthesis method also plays a critical role in determining photocatalytic performance. For instance, Sr_3_Ti_2_O_7_ prepared via the polymerized complex method exhibits higher photocatalytic activity compared with samples synthesized by conventional solid-state reactions, primarily due to improved crystallinity, smaller particle size, and better control of defect distribution [335]. Furthermore, heterostructure engineering, including Z-scheme systems and type-II heterojunctions, has been explored to enhance charge separation and extend light absorption. For example, a heterostructure composed of La_2_CuO_4_ and Cu_2_O has been reported to exhibit enhanced photocatalytic activity, where La_2_CuO_4_ mainly contributes to water reduction while Cu_2_O promotes efficient electron–hole separation under light irradiation.

Recent advances in layered perovskites further demonstrate that morphology engineering plays a crucial role in enhancing photocatalytic performance. As summarized by Hu et al., modified layered perovskites, particularly exfoliated nanosheets, exhibit hydrogen evolution rates several times higher than those of their bulk counterparts under visible-light irradiation. Exfoliation into ultrathin nanosheets substantially increases the specific surface area while eliminating long bulk carrier-diffusion pathways. In addition, the interlayer galleries and exposed nanosheet surfaces provide spatially separated reduction and oxidation sites, thereby suppressing electron–hole recombination. Restacking and hybrid assembly strategies further optimize the electronic band structure and light-harvesting capability. Consequently, nanosheet-based layered perovskites achieve superior charge separation and enhanced photocatalytic water-splitting activity owing to their high surface area and shortened carrier-migration distances [341]. Layered perovskites, such as La_2_Ti_2_O_7_, have demonstrated considerable potential for photocatalytic water splitting due to their anisotropic crystal structure and favourable charge-transport properties. Kim et al. reported that, under UV irradiation, the best NiO-loaded La_2_Ti_2_O_7_ sample prepared using TiO_2_-P25 achieved a hydrogen evolution rate of 788 µmol g^−1^ h^−1^. The photocatalytic activity depended strongly on the synthesis route, with wet-ground precursors yielding a significantly higher H_2_ evolution rate (473 µmol g^−1^ h^−1^) than dry-ground precursors (150 µmol g^−1^ h^−1^). Wet grinding produced uniform 1–2 µm particles with higher crystallinity and phase purity, whereas dry grinding resulted in irregular 1–5 µm particles containing residual La_2_O_3_. The morphology of the La_2_O_3_ precursor largely determined the final microstructure, with finer precursor particles promoting higher crystallinity and larger surface area. Likewise, the use of fine TiO_2_ precursors, particularly P25, facilitated the solid-state reaction and enhanced phase purity. These structural and morphological improvements directly translated into superior photocatalytic water-splitting performance [342].

Ruddlesden–Popper perovskites possess highly tunable layered structures that can facilitate charge separation and provide numerous opportunities for compositional engineering. However, despite these attractive structural characteristics, their application in photocatalytic overall water splitting remains at an early stage. Most reported studies have focused on improving individual photocatalytic half-reactions or investigating fundamental charge-transfer mechanisms rather than achieving efficient overall water splitting [333]. Compared with oxide and oxynitride perovskites, their photocatalytic efficiencies remain relatively modest, highlighting the need for improved band-gap engineering, cocatalyst optimization, and long-term stability studies before their full potential can be assessed [343]. Table 5 summarizes the majority of Ruddlesden–Popper perovskite photocatalysts that have been reported and investigated for photocatalytic water splitting [344].

Overall, as discussed above, most of these materials have primarily been investigated for photocatalytic half-reactions rather than overall water splitting. This observation may indicate that their band edge positions are not suitably aligned to simultaneously drive both HER and the OER reactions. Consequently, RP perovskites as candidates to achieve photocatalytic performances comparable to those reported for SrTiO_3_ in the study by Takata and co-workers [21] may not be the most appropriate strategy.

## 8. Aurivillius Perovskite

Aurivillius-type perovskites constitute an important class of layered oxide materials that have attracted considerable attention for photocatalytic applications, including photocatalytic water splitting. These compounds generally follow the chemical formula Bi_2_A_n−1_B_n_O_3n+3_, where A represents a large cation (such as Ca, Sr, Ba, or Na), B is typically a transition metal cation (e.g., Ti, Nb, Ta, or W), and n denotes the number of perovskite-like octahedral layers within the structure. Structurally, Aurivillius phases consist of alternating layers of (Bi_2_O_2_)^2+^ sheets and perovskite-like slabs (A_n−1_B_n_O_3n+1_)^2−^ stacked along the crystallographic c-axis. The perovskite blocks are composed of corner-sharing BO_6_ octahedra, similar to conventional perovskites, while the bismuth oxide layers act as structural separators. This layered architecture results in strong structural anisotropy and plays a crucial role in determining the electronic and photocatalytic properties of these materials [348,349].

Aurivillius oxides have attracted attention as photocatalysts for water splitting owing to their favorable electronic structure and layered architecture. In these materials, the conduction band is typically derived from transition-metal d orbitals (e.g., Nb^5+^, Ta^5+^, or W^6+^), while the valence band mainly consists of O 2p orbitals hybridized with Bi 6s/6p states. The presence of Bi raises the valence-band energy through orbital hybridization, which can enhance visible-light absorption compared with conventional oxide perovskites. A key advantage of Aurivillius phases is their intrinsic charge-separation capability arising from their layered structure. Electrons preferentially migrate through the perovskite slabs containing BO_6_ octahedra, whereas holes tend to accumulate in the (Bi_2_O_2_) layers. This spatial separation suppresses electron–hole recombination and promotes surface redox reactions, with electrons driving hydrogen evolution and holes facilitating water oxidation [350,351]. Additionally, internal electric fields generated by the asymmetric layered structure can further enhance directional charge transport. The two-dimensional nature of Aurivillius compounds also favors anisotropic morphologies, such as nanosheets, which shorten carrier diffusion paths and increase the density of active surface sites [352,353].

Strategies have been developed to further enhance the photocatalytic performance of Aurivillius-type materials. For example, triclinic Bi_2_CrO_6_ exhibits a broad light absorption range up to approximately 650 nm with a direct band gap of about 1.86 eV. Theoretical studies indicate that this material possesses a relatively large charge-carrier effective mass and can exhibit enhanced photocatalytic performance when coupled with a second photocatalyst such as SrTiO_3_, enabling overall water splitting in a heterostructured system [354]. Doping strategies have also been explored to improve photocatalytic activity [355]. For instance, Cr/Nb co-doping of Bi_3_TiNbO_9_ has been reported to significantly enhance hydrogen production, yielding approximately twice the hydrogen evolution compared with the undoped material [356]. Similarly, gradient tungsten doping in Bi_3_TiNbO_9_ can improve photocatalytic activity by introducing an additional built-in electric field along the c-axis and simultaneously enhancing the depolarization field within the layers along the a-axis due to strengthened structural distortion [357]. Surface engineering represents another effective approach for improving photocatalytic performance. For example, the surface layer of Bi_3_TiNbO_9_ can be selectively modified through an acid-etching strategy, which improves its resistance to photocorrosion. In this system, efficient electron transfer occurs via surface trapping states, while effective hole transfer is promoted by a surface electric field, leading to spatial separation of photogenerated charge carriers. Moreover, Bi_3_TiNbO_9_ forms a relatively small Schottky barrier when interfaced with cocatalysts, facilitating electron transfer to the cocatalyst and enabling efficient and stable overall water splitting [358]. Crystal facet engineering has also been shown to influence photocatalytic performance. In particular, the ratio of exposed {001} and {110} facets in Bi_3_TiNbO_9_ can play an important role in determining the activity toward HER and OER reactions. Additionally, anion-induced surface structure transformation has been employed to tailor the interface between Bi_3_TiNbO_9_ and cocatalysts [357]. For example, an ultrathin Bi_2_MoO_6_ heterolayer can be grown in situ on Bi_3_TiNbO_9_ nanosheets, creating a favorable interface with Rh cocatalysts. This engineered interface lowers the interfacial energy barrier and promotes the transfer of photogenerated electrons from Bi_3_TiNbO_9_ to Bi_2_MoO_6_ and subsequently to the Rh cocatalyst, thereby enhancing photocatalytic efficiency [359].

Aurivillius perovskites benefit from their layered crystal structure and spontaneous polarization, which can promote charge separation and suppress electron–hole recombination [360]. These characteristics make them attractive candidates for photocatalytic applications. Nevertheless, despite these intrinsic advantages, their photocatalytic activity remains relatively limited, and convincing demonstrations of efficient overall water splitting are still lacking [343]. Their electronic structures often require further optimization through elemental substitution or heterostructure engineering to achieve suitable band gaps and band-edge positions. Table 6 shows the reported Aurivillius-type perovskite for photocatalytic water-splitting application.

## 9. Dion–Jacobson Perovskite

Dion–Jacobson (DJ) perovskites represent an important class of layered perovskite oxides that have attracted increasing attention in photocatalysis, particularly for photocatalytic water splitting. These materials typically follow the general formula A′A_n−1_B_n_O_3n+1_, where A′ is a large interlayer cation (commonly an alkali or alkaline-earth metal such as Cs, Rb, or K), A is typically an alkaline-earth or rare-earth metal, B is a transition metal (e.g., Ti, Nb, or Ta), and n denotes the number of perovskite layers in the structure. Structurally, Dion–Jacobson phases consist of perovskite-like slabs composed of corner-sharing BO_6_ octahedra, separated by a single layer of interlayer cations [365]. Unlike Ruddlesden–Popper phases, which contain rock-salt layers, DJ structures feature direct bonding between adjacent perovskite slabs through the interlayer cations, resulting in stronger interlayer interactions. This structural configuration provides several advantages for photocatalytic applications. The absence of rock-salt layers leads to shorter interlayer distances and stronger electrostatic interactions, which can improve charge transport between the perovskite slabs [366]. In addition, the interlayer space can accommodate ion exchange and structural modifications, enabling tunability of the electronic structure and catalytic properties. These features make Dion–Jacobson perovskites particularly attractive platforms for designing layered photocatalysts [367].

From an electronic perspective, the band structure of Dion–Jacobson oxides is similar to that of many oxide perovskites. The valence band is mainly composed of O 2p orbitals, while the conduction band originates primarily from the d orbitals of the transition metal cations (e.g., Nb 4d, Ta 5d, or Ti 3d) [368]. Consequently, many DJ-type oxides exhibit wide band gaps, typically in the range of 3.0–3.7 eV and are mainly active under ultraviolet irradiation. This trend is clearly reflected in the band gap values summarized in Table 7. Representative photocatalysts include layered niobates and tantalates such as CsCa_2_Nb_3_O_10_, RbLaNb_2_O_7_, and KCa_2_Nb_3_O_10_, which have been investigated for hydrogen evolution reactions in photocatalytic systems [369,370,371]. In water-splitting systems, Dion–Jacobson perovskites are most used for HER reactions in the presence of suitable cocatalysts such as Pt, Rh, or NiO, which facilitate proton reduction and suppress charge recombination [372,373].

Dion–Jacobson perovskites provide additional flexibility through their layered architecture and tunable interlayer chemistry, offering opportunities to tailor charge transport and surface reaction pathways [374]. However, research on these materials for photocatalytic overall water splitting remains relatively limited compared with conventional oxide and oxynitride perovskites. To date, all reported photocatalysts belonging to this family possess relatively large band gaps, preventing efficient utilization of the visible region of the solar spectrum. Moreover, although their structural tunability represents a clear advantage, relatively few studies have demonstrated high photocatalytic efficiencies, making it difficult to establish general structure–property relationships [375]. Table 7 illustrates a summary of reported Dion-Jacobson photocatalysts.

**Table 7:** Summary of Dion–Jacobson perovskite photocatalytic systems for water splitting.

Perovskite, (Band Gap [eV])	Incident Light	Solution	Cocatalyst	H_2_^1^	O_2_^1^	Ref.
HCa_2_Nb_3_O_10_ (3.4)	125 W Hg lamp λ > 220 nm	1 mol.% aqueous solutions of D-glucose and D-xylose	1 wt.% Pt	2000		[376]
H_2_LaNb_2_O_7_ (3.4)	100 W Hg lamp	10 vol.% methanol	Pt	1628		[372]
KCa_2_Nb_3_O_10_ (3.6)	300 W Xe lamp λ > 300 nm	0.01 M NaI	1.3 wt.% Pt	220	82	[371]
RbLaNb_2_O_7_ (3.3)	UV lamp, λ > 230 nm	20 vol.% methanol	1 wt.% Pt	373		[370]
AgLaNb_2_O_7_ (3)	UV lamp, λ > 230 nm	“	“	1806		“
	500 W halogen lamp	16 vol.% methanol	NiO_x_	144		[373]
HCa_2_Nb_3_O_10_ (3.5)	125 W Hg lamp λ > 220 nm	1 mol.% methanol	1 wt.% Pt	2083		[377]
KCa_2_Nb_3_O_10_ (3.5)	“	“	“	250		“
KCa_2_Ta_3_O_10_ (3.4)	300 W Xe lamp λ > 420 nm	20 vol.% methanol	1 wt.% Pt	0		[378]
	“	“	1 wt.% Pt/gC_3_N_4_	215		“
LiCa_2_Ta_3_O_10_ (4.2)	400 W Hg lamp	-	NiO_x_	1780	970	[369]
NaCa_2_Ta_3_O_10_ (4.3)	“	-	“	1465	810	“
	“	-	0.5 wt.% NiO_x_	1406	703	[379]
KCa_2_Ta_3_O_10_ (4.2)	“	-	“	870	400	[369,380]
RbCa_2_Ta_3_O_10_ (3.9)	“	-	“	900	455	[369]
CsCa_2_Ta_3_O_10_ (4.1)	“	-	“	445	202	“
	“	-	“	1062	531	[379]
HCa_2_Ta_3_O_10_ (4.2)	“	-	“	937	468	“

^1^ The units are [μmol g^−1^ h^−1^].

## 10. Roadmap for the Development of High-Performance Perovskite Photocatalysts

The studies discussed throughout this review indicate that several perovskite materials already possess the fundamental characteristics required for photocatalytic water splitting. Successful UV-driven overall water splitting, half-reaction activity, suitable band gap and Mott–Schottky analysis demonstrate that these materials have the intrinsic potential for overall water splitting. Therefore, future research should focus less on discovering entirely new perovskite compositions and more on engineering promising existing materials.

Among the investigated materials, PbTiO_3_ from the ABO_3_ perovskite family and several oxynitride perovskites possess narrower band gaps than SrTiO_3_ while maintaining suitable band positions for water splitting. In particular, LaTaON_2_, BaTaO_2_N, CaTaO_2_N, and LaTiO_2_N, with band gaps of approximately 2.0, 1.8, 2.2, and 2.1 eV, respectively, have been reported to perform overall water splitting without sacrificial agents, as well as half water-splitting reactions in the presence of sacrificial agents (Table 4).

For example, Qi et al. [233] reported overall water splitting using Na/Mo-codoped PbTiO_3_ in a Z-scheme system combined with RhCrO_x_/PbTiO_3_. However, the dopant type, doping level, and cocatalyst deposition method differ from those reported by Takata et al. For LaTiO_2_N, Wang et al. [313] employed the same cocatalyst deposition strategy as Takata et al. and achieved remarkable overall water-splitting activity under simulated AM 1.5 G irradiation using pure water, although no doping was introduced. For BaTaO_2_N, Li et al. [294] employed a similar high-temperature synthesis method but used different cocatalysts and deposition methods, together with a different dopant concentration that was approximately five times higher than that reported by Takata et al. Nevertheless, this photocatalyst successfully performed visible-light-driven overall water splitting in pure water. Similarly, Xu et al. [314] synthesized CaTaO_2_N using a high-temperature method but without doping, while the cocatalysts were deposited by impregnation rather than photodeposition, which can affect cocatalyst dispersion. Likewise, Pan et al. [61] investigated LaTaON_2_ using different dopant concentrations and cocatalyst deposition methods, yet still demonstrated overall water splitting in pure water.

Based on these observations, the following roadmap is proposed. Starting with the most promising candidates, namely PbTiO_3_, LaTaON_2_, BaTaO_2_N, CaTaO_2_N, and LaTiO_2_N, the first step should be the synthesis of highly crystalline materials using high-temperature synthesis methods. During the synthesis, these materials can be modified by introducing very low concentrations of common dopants such as Al or Mg, or less commonly investigated dopants such as Ta, La, Fe, or W. This low-level doping strategy is well established in semiconductor engineering, where small amounts of dopants can significantly improve charge carrier mobility without altering the crystal structure. In contrast, excessive doping may result in the formation of new phases, reduce crystallinity, or even change the perovskite structure. Cocatalyst deposition is another critical factor, and it is recommended to employ the same photodeposition strategy reported by Takata et al., which has demonstrated exceptional performance for SrTiO_3_.

Several other oxynitride perovskites despite their smaller band gap have also been reported to perform half water-splitting reactions; however, they have either never been evaluated for overall water splitting or have only been investigated as part of Z-scheme systems. These materials should also be systematically evaluated. As a first step, the undoped perovskites decorated with cocatalyst should be tested for overall water splitting under UV irradiation. If measurable activity is observed, systematic optimization of the dopant type, dopant concentration, and cocatalyst deposition strategy can then be carried out and tried with visible light. Following this approach, it may be possible to achieve significantly higher photocatalytic activities for overall water splitting.

## 11. Conclusions and Future Perspectives

The first major challenge in achieving practical solar water splitting is the identification of a photocatalyst that simultaneously exhibits efficient visible-light absorption, appropriate conduction- and valence-band positions for overall water splitting, and long-term stability. Most reported photocatalysts either possess suitable band-edge positions but absorb predominantly ultraviolet light or exhibit visible-light absorption at the expense of insufficient redox potential. Once an appropriate photocatalyst has been identified, the remaining bottlenecks involve maximizing the utilization of photogenerated charge carriers. These include suppressing electron–hole recombination, extending charge-carrier lifetimes through defect engineering and improved crystallinity, and optimizing surface reaction kinetics through rational cocatalyst deposition. Efficient cocatalysts are essential for accelerating the sluggish multielectron oxygen and hydrogen evolution reactions while minimizing interfacial recombination losses. Simultaneously, maintaining long-term structural and photochemical stability under operating conditions remains a prerequisite for practical application.

The development of Al-doped SrTiO_3_ modified with Rh/Cr_2_O_3_ and CoOOH cocatalysts demonstrated that these challenges can be largely overcome through an integrated materials-design strategy, resulting in an apparent quantum efficiency approaching unity under ultraviolet irradiation. This achievement indicates that the fundamental limitations associated with charge separation and surface reaction kinetics can be addressed when the semiconductor, dopant concentration, crystallinity, and cocatalyst architecture are simultaneously optimized. However, the major limitation of this system is no longer charge utilization but its wide band gap, which restricts light absorption to the ultraviolet region.

In this review, by presenting the perovskites used in photocatalytic water splitting, we sought to answer the following question: Are there any perovskites other than SrTiO_3_ that are capable of absorbing a broader range of the solar spectrum so that, if the same engineering strategy is applied, they could potentially achieve quantum efficiencies comparable to those of SrTiO_3_. Different classes of perovskites, including simple ABO_3_ oxides, double oxides, oxynitrides, Aurivillius, Dion–Jacobson, and Ruddlesden–Popper perovskites, were reviewed. Among the oxide-based perovskites, only PbTiO_3_ has been reported in the literature to achieve overall water splitting. The remaining oxide perovskites either possess excessively wide band gaps or unfavorable band-edge positions, allowing only one of the half-reactions to occur, and therefore no overall water splitting has been reported. However, the situation is considerably more promising for oxynitride perovskites. Several oxynitride compounds, including LaTaON_2_, BaTaO_2_N, CaTaO_2_N, and LaTiO_2_N, have been reported to achieve overall water splitting under visible-light irradiation. To date, the design strategy that enabled SrTiO_3_ to achieve near-unity quantum efficiency has not yet been systematically applied to these materials. Most published studies investigate these perovskites either in their pristine form or employ relatively high dopant concentrations, different synthesis protocols, or cocatalyst deposition methods that differ substantially from the optimized approach established for SrTiO_3_. Consequently, it remains unclear whether the remarkable performance achieved with SrTiO_3_ can be replicated in visible-light-responsive perovskites.

Therefore, future research should move beyond the continued discovery of new perovskite compositions and instead focus on systematically transferring and validating the successful design principles established for SrTiO_3_ to the most promising visible-light-responsive perovskites. Such studies are essential to determine whether PbTiO_3_ and oxynitride perovskites can combine broad solar-light harvesting with the near-unity charge-utilization efficiency already demonstrated for SrTiO_3_, thereby providing a realistic pathway toward practical solar hydrogen production.

## Figures and Tables

**Table 1:** Simple ABO_3_-based perovskites reported in organic and pollutant photocatalytic decomposition.

Perovskite		Bad Gap [eV]	Organic Decomposition
MFeO_3_	Ce	2	methylene blue [137], pesticides [138], tetracycline and dye [139], gentian violet [140]
	Nd	2.4	organic dye and antibiotic [141], tetracycline [142], methyl orange [143]
	Sm	2.3	rhodamine-B [144], orange II dye and the tetracycline hydrochloride [145]
	Pr	2.1	methyl orange [146], erythrosine and green malachite [147], tetracycline [148]
	Eu	2.3	methyl orange [149], congo red [150], various organic dyes and fuchsine [151]
	Gd	2.3	methylene blue [152], rhodamine B [153], norfloxacin [154]
	Y	2	methyl orange and 4-chlorophenol [155], Rhodamine B [156]
	Sr	1.8	acid red B [157], nitrobenzene [158], chloramphenicol and crystal violet [159]
	Ga	2.1	methyl violet and methylene blue [160], rhodamine B [161]
MCrO_3_	La	3.3	methylene blue [162], crystal violet (CV) dye [163]
	Ce	1.3	fast green dye [164]
	Gd	2.7	congo red dye [165]
	Sm	2.7	rhodamine 6G dye [166]
	Bi	2.5	tetracycline hydrochloride, metronidazole, azithromycin, and cephalexin [167]
MBiO_3_	Ba	2	malachite Green dye [168], phenol [169], benzene [170]
	Ca	2.7	ciprofloxacin and tetracycline [171]
	K	2.1	rhodamine B, crystal violet, methylene blue and phenol [172]
	Li	1.8	methylene blue [173], rhodamine B [174]
MVO_3_	La	3.3	methylene blue [175]
	Bi	2.2	amaranth dye [176]
MCeO_3_	Sr	2.8	methylene blue [177], titan yellow dye [178]
MNbO_3_	Ag	2.8	sulfamethoxazole [179], rhodamine B [180], methylene blue (MB) [181]
MCoO_3_	La	1.3	indigo carmine dye, methyl orange, Congo Red, methylene blue, Rhodamine B [182]
MNiO_3_	Ce	2	rhodamine B, oxytetracycline [183], orange-G (OG) dye [184]
	La	2.6	methyl orange [185], tetracycline [186], bisphenol A & reduction of Cr(VI) [187]
MMnO_3_	Ce	1.9	methylene blue & methylene orange [188], tetracycline hydrochloride & methylene blue [189]
	La	1.4	methyl orange [190], tetracycline [191], acid red A [192]
	Gd	1.2	methyl orange [193], rhodamine B [194]

**Table 2:** Summary of PbTiO_3_-based photocatalytic systems for water splitting.

Catalyst	Incident Light	Solution	Cocatalyst	H_2_^1^	O_2_^1^	Ref.
PbTiO_3_	1000 W Xe lamp, λ > 420 nm	20 vol.% methanol	1 wt.% Pt	27		[237]
	“	0.1 M AgNO_3_	1 wt.% RuO_2_		180	
PbTiO_3_	Visible light	10 vol.% glycerol	0.8 wt.% Pt	380		[253]
PbTiO_3_	300 W Xe lamp	10 vol.% triethanolamine	1 wt.% Pt, Au, Ag/MnO_x_	75		[254]
PbTiO_3_	300 W Xe lamp	20 vol.% methanol	Pt(Au), MnO_x_	160		[255]
Cu-Doped PbTiO_3_	125 W Hg lamp, λ > 400 nm	10 vol.% methanol	-	900	-	[256]
Na/Mo co-doped PbTiO_3_	300 W Xe lamp λ > 420 nm	2 mM FeCl_3_	1 wt.% RuO_2_ or RhCrO_x_	36	18	[233]
PbTiO_3_ modulated by oxygen vacancy	300 W Xe lamp	-	Rh/Cr_2_O_3_ or Au/MnO_x_	33	16	[235]
B-rGO/PbTiO_3_	125 W medium pressure Hg, λ > 420 nm	10 vol.% methanol	-	150	-	[257]
PbTiO_3_-TiO_2_	500 W high-pressure mercury lamp	20 vol.% methanol	1 wt.% Pt	210		[251]
rGO/PbTiO_3_	500 W Xe lamp	10 vol.% triethanolamine	-	5		[258]
MoS_2_/PbTiO_3_	300 W Xe lamp, λ > 420 nm	0.05 g L^−1^, tetracycline	1 wt.% Pt	45		[236]
TiO_2_/PbTiO_3_	300 W Xe lamp	10 vol.% triethanolamine	Pt	410		[252]
	“	AgNO_3_	MnO_x_		16	
	300 W Xe lamp, λ > 420 nm	10 vol.% triethanolamine	Pt	40		
	“	AgNO_3_	MnO_x_		5	
g-C_3_N_4_/PbTiO_3_	300 W Xe lamp, λ > 420 nm	10 vol % triethanolamine	2 wt.% Pt	340		[259]
g-C_3_N_4_/PbTiO_3_	300 W Xe lamp	50 vol.% ethanol	2 wt.% Pt	1580		[260]
SiO_2_(ALD)/PbTiO_3_	300 W Xe lamp	20 vol.% methanol	Pt	450		[239]
SrTiO_3_/PbTiO_3_	300 W Xe lamp, λ > 300 nm	-	0.25 wt.% Rh/Cr_2_O_3_	800	360	[247]
SrTiO_3_/PbTiO_3_	300 W Xe lamp	-	Rh, CrO_x_, CoOOH	1100	600	[232]
Cu@PbTiO_3_/g-C_3_N_4_	350 W metal halid lamp	5 vol.% triethanolamine	Pt	550		[240]
LaCrO_3/_PbTiO_3_	150 W Xe lamp, λ > 420 nm	10 vol.% methanol	Pt, Au/MnO_x_	170		[261]
CdS/PbTiO_3/_TiO_2_	300 W Xe lamp, λ > 420 nm	Na_2_S & Na_2_SO_3_	Pt, Au/MnO_x_	1800		[262]
CuP/PbTiO_3_	300 W Xe lamp, λ > 420 nm	10 vol.% methanol	-	90		[263]

^1^ The units are [μmol g^−1^ h^−1^].

**Table 3:** Reported double perovskite photocatalysts for water splitting.

Perovskite (Band Gap, [eV])	Catalyst	Incident Light	Solution	Cocatalyst	H_2_^1^	O_2_^1^	Ref.
Ba_2_FeNbO_6_ (2.3)	SrTiO_3_-Ba_2_FeNbO_6_	300 W Xe lamp λ > 420 nm	25 vol.% methanol	1 wt.% Pt	5		[275]
Sr_2_NiWO_6_ (2.9)	Sr_2_NiWO_6_	“	0.02 M Na_2_S_2_O_8_	1 wt.% Pt, RuO_2_		420	[276]
Sr_2_CoWO_6_ (1.7)	Sr_2_CoWO_6_	300 W Xe lamp λ > 420 nm	Fe (NO_3_)_3_, FeCl_3_, AgNO_3_	Pt, Rh		188	[277]
		“	0.02 M Na_2_S + Na_2_SO_3_	-	30		“
Sr_2_FeNbO_6_ (2.1)	Sr_2_FeNbO_6_	450 W Xe lamp λ > 420 nm	20 vol.% methanol	0.2 wt% Pt	135		[278]
	W-doped Sr_2_FeNbO_6_	“	“	“.	110		[272]
La_2_FeTiO_6_ (2)	La_2_FeTiO_6_	300 W Xe lamp λ > 420 nm	0.1 M Na_2_S + Na_2_SO_3_	0.5 wt.% Pt, 1 wt.% RuO_2_	7		[269]
		“	0.02 M AgNO_3_	“		60	“
Sr_2_FeTaO_6_ (2.3)	Sr_2_FeTaO_6_	500 W Hg lamp λ > 400 nm	0.05 M Na_2_SO_3_	1 wt.% Pt	100		[270]
	“	300 W Xe lamp λ > 420 nm	15 vol.% TEOA	0.5 wt.% Pt	700		[273]
	Sr_2_FeTaO_6_/NaTaO_3_	“	“	“	1300		“
	Sr_2_FeTaO_6_	300 W Xe lamp λ > 420 nm	20 vol.% TEOA	2 wt.% Pt/NiO	2500		[279]
Sr_2_CoTaO_6_ (2.7)	Sr_2_CoTaO_6_	300 W Xe lamp λ > 420 nm	AgNO_3_ + La_2_O_3_	0.5 wt.% RuO_2_		60	[271]
	“	“	Na_2_S + Na_2_SO_3_	“	4		“

^1^ The units are [μmol g^−1^ h^−1^].

**Table 4:** Reported oxynitrides photocatalysts for water splitting.

Perovskite (Band Gap [eV])	Catalyst	Incident Light	Solution	Cocatalyst	H_2_^1^	O_2_^1^	Ref.
BaNbO_2_N (1.7)	Mg-doped BaNbO_2_N	300 W Xe lamp λ > 420 nm	0.05 M AgNO_3_ + 2 gL^−1^ La_2_O_3_	2 wt.% CoO_x_		200	[62]
	Mg-doped BaNbO_2_N + Ru/SrTiO_3_:Rh	solar insolation (AM 1.5 G)	0.002 M FeCl_3_	1 wt.% CoO_x_	9	4	“
	BaNbO_2_N	300 W Xe lamp λ > 410 nm	0.05 M AgNO_3_ + 1 gL^−1^ La_2_O_3_	2 wt.% CoO_x_		300	[315]
	“	“	80 vol.% CH_3_OH	1 wt.% Pt	0.2		“
	Ca-doped BaNbO_2_N	300 W Xe lamp λ > 420 nm	0.05 M AgNO_3_ + 1 gL^−1^ La_2_O_3_	2 wt.% CoO_x_		90	[305]
	BaNbO_2_N	“	0.05 M AgNO_3_ + 2 gL^−1^ La_2_O_3_	1 wt.% CoO_x_		850	[316]
SrNbO_2_N (2.2)	SrNbO_2_N	“	0.05 M AgNO_3_	2 wt.% CoO_x_		730	[307]
	With 2nd photocatalyst 0.5% wt Ru/SrTiO_3_:Rh	simulated sunlight (AM 1.5 G filter)	0.002 M Fe^2+^/Fe^3+^ redox shuttle	2 wt.% CoO_x_	34	17	“
	Ta-doped SrNbO_2_N	300 W Xe lamp λ > 420 nm	20 vol.% TEOA	1 wt.% Pt	88		[306]
	Mg-doped SrNbO_2_N	300 W Xe lamp λ > 400 nm	0.05 M AgNO_3_ + 2 gL^−1^ La_2_O_3_	1 wt.% CoO_x_		650	[301]
CaNbO_2_N (2)	Mg-doped CaNbO_2_N	300 W Xe lamp λ > 420 nm	0.05 M AgNO_3_ + 2 gL^−1^ La_2_O_3_	2 wt.% CoO_x_		1000	[300]
	CaNbO_2_N	“	10 vol.% Methanol	1 wt.% Pt	10		[308]
	“	“	0.01 M AgNO_3_ + 2 gL^−1^ La_2_O_3_	-		22.5	“
LaNbON_2_ (1.7)	g-C_3_N_4_/LaNbON_2_	300 W Xe lamp (AM 1.5 G filter)	0.05 M AgNO_3_	-		37	[317]
	“	“	-	1 wt.% Pt, 0.1 wt.% CoO_x_	100	50	“
	LaNbON_2_	300 W Xe lamp (AM 1.5 G filter)	0.02 M AgNO_3_ +2 gL^−1^ La_2_O_3_	2 wt.% CoO_x_		34	[318]
LaTaON_2_ (2)	LaTaON_2_	300 W Xe lamp λ > 420 nm	0.05 M AgNO_3_	CoO_x_		240	[319]
	With 2nd photocatalyst SrTiO_3_:Rh	“	0.002 M Fe^2+^/Fe^3+^ redox shuttle	CoO_x_	40	20	“
	LaTaON_2_	“	20 vol.% ethanol	0.15 wt.% Pt, 0.25 wt.% Ru	125		[320]
	TiO_2_/Mg-doped LaTaON_2_	300 W Xe lamp λ > 300 nm	-	3 wt.% CoO_x_/RhCrO_x_	7	1.8	[61]
	LaTaON_2_	300 W Xe lamp λ > 420 nm	0.05 M AgNO_3_ + 2 gL^−1^ La_2_O_3_	2 wt.% CoO_x_		1466	[313]
	“	“	0.2 M acid ascorbic	1 wt.% Pt	270		“
	“	Simulated AM 1.5 G illumination (100 mW cm^−2^)	-	0.1 wt.% Rh/CrO_x_,0.05 wt.% CoOOH	13	6.5	“
	LaTaON_2_-SrZrO_3_	300 W Xe lamp λ > 420 nm	0.05 M AgNO_3_ + 2 gL^−1^ La_2_O_3_	2 wt.% CoO_x_		132	[299]
	LaTaON_2_-SrZrO_3_ with 2nd photocatalyst Rh:SrTiO_3_	“	0.002 M FeCl_3_	2 wt.% CoO_x_	21	10	“
	Al-doped LaTaON_2_	“	0.05 M Na_2_SO_3_	1 wt.% Pt			[321]
	“	“	0.05 M AgNO_3_	2 wt.% CoO_x_		160	“
	With 2nd photocatalyst Rh:SrTiO_3_	300 W Xe lamp with AM 1.5 G	0.002 M FeCl_3_	2 wt.% CoO_x_	18	10	“
BaTaO_2_N (1.8)	Mg-doped BaTaO_2_N	300 W Xe lamp 800 > λ > 420 nm	-	2 wt.% Rh/2 wt.% Cr_2_O_3_, 0.3 wt.% IrO_2_	44	20	[294]
	“	300 W Xe lamp, AM 1.5 G	-	“	4.2	2	“
	BaTaO_2_N-BaZrO_3_	300 W Xe lamp λ > 420 nm	0.004 M NaI	0.3 wt.% Pt	18.75		[287]
	BaTaO_2_N-BaZrO_3_ With 2nd photocatalyst Pt/WO_3_	“	0.001 M NaI	“	13.75	6.25	“
	BaTaO_2_N	“	-	2 wt.% Rh, 1 wt.% Cr_2_O_3_ and 0.3 wt% IrO_2_	8	3.5	[296]
	“	300 W Xe lamp AM 1.5 G solar	-	“	1.3	0.6	“
	“	300 W Xe lamp λ > 420 nm	20 vol.% methanol	0.3 wt.% Pt	410		[322]
	NH_3_-BaTaO_2_N	“	0.05 M AgNO_3_ + 2 gL^−1^ La_2_O_3_	2 wt.% CoO_x_		800	[310]
	With 2nd photocatalyst Pt/CoO_x_/SrTaO_2_N	“	0.025 M Na_3_PO_4_ buffer (pH 8), 0.005 M K_3_[Fe(CN)_6_].	0.3 wt.% Pt/0.9 wt.% Cr_2_O_3_	30	15	[295]
	“	“	0.025 M Na_3_PO_4_ buffer (pH 8), 0.001 M [Co(bpy)_3_]^3+/2+^	“	20	10	“
SrTaO_2_N (2)	SrTaO_2_N	300 W Xe lamp λ > 420 nm, with a water filter	20 vol.% methanol	0.3 wt.% Pt	400		[322]
	“	“	0.005 M NaI	“	1.4	0	“
	SrTaO_2_N with 2nd photocatalyst Ir-CoFeO_x/_BiVO_4_	solar simulator (AM 1.5 G)	0.025 M Na_3_PO_4_ + 0.005 M K_4_[Fe(CN)_6_]	Cr_2_O_3_/Pt/Ir	120	50	[309]
	SrTaO_2_N	300 W Xe lamp λ > 420 nm	13 vol.% methanol	“	1600		“
	“	“	0.02 M AgNO_3_ + 0.6 gL^−1^ La_2_O_3_	CoO_x_		1142	“
	SrTaO_2_N	300 W Xe lamp λ > 420 nm	0.05 M AgNO_3_ + 2 gL^−1^ La_2_O_3_	CoO_x_		600	[288]
	With 2nd photocatalyst SrTiO_3_:Rh	“	0.02 M Fe^2+^/Fe^3+^ redox couple	2 wt.% CoO_x_	11.7	5.7	“
	Mg-doped SrTaO_2_N	300 W Xe lamp λ > 420 nm	0.025 M phosphate buffer (pH = 6) + 0.01 K_4_[Fe(CN)_6_]	0.7 wt.% Pt/1.05 wt.% Cr_2_O_3_	3500		[303]
	Mg-SrTaO_2_N With 2nd photocatalyst Au-CoFeO_x_/BiVO_4_	“	0.025 M sodium potassium phosphate buffer (pH = 6.0) + 0.01 M K_4_[Fe(CN)_6_]	“	275	125	“
	SrO- SrTaO_2_N	“	0.025 M AgNO_3_ + 5 gL^−1^ La_2_O_3_	2 wt.% CoO_x_		80	[323]
CaTaO_2_N (2.2)	CaTaO_2_N	300 W Xe lamp λ > 300 nm	-	RhCrO_y_	5.5	2.75	[314]
	“	300 W Xe lamp λ > 420 nm	-	“	0.7	0.35	“
	CaTaO_2_N–CaZrO_3_	“	11 vol.% formic acid	1 wt.% Pt	52.4		[324]
	“	“	“	“	23.1		“
	Ta_3_N_5_/CaTaO_2_N	300 W Xe lamp λ > 420 nm	20 vol.% methanol	0.3 wt.% Pt	51.3		[325]
	“	“	0.001 M NaI	“	34	16.7	“
	Ta_3_N_5_/CaTaO_2_N with 2nd photocatalyst PtO_x_/WO_3_	“	20 vol.% methanol	“	5.3		“
	“	“	0.001 M NaI	“	3	1.4	“
	Ta_3_N_5_/Bi/CaTaO_2_N	300 W Xe lamp λ > 420 nm	20 vol.% Methanol	1 wt.% Pt	290		[298]
	“	“	0.005 M AgNO_3_ + 1 gL^−1^ La_2_O_3_	2 wt.% CoO_x_		3600	“
	Ta_3_N_5_/Bi/CaTaO_2_N	“	-	1 wt.% RhCrO_x_	44	21	“
	CaTaO_2_N	“	-	“	3.5	1.75	“
LaTiO_2_N (2.1)	LaTiO_2_N	300 W Xe lamp λ > 420 nm	0.05 M AgNO_3_	2 wt.% CoO_x_		1670	[313]
	“	Simulated AM 1.5 G illumination	-	0.1 wt.% RhCrO_y_, 0.05 wt.% CoOOH	13.3	6.7	“
	LaTiO_2_N nanosheets	300 W Xe lamp λ > 420 nm	-	0.5 wt.% RhCrO_y_ 1 wt.% Ti oxyhydroxide layer	13.6	6.7	[289]
	Mg-doped LaTiO_2_N	300 W Xe lamp λ > 420 nm	0.05 M AgNO_3_ + 2 gL^−1^ La_2_O_3_	2 wt.% CoO_x_		1400	[304]
	Mg/LaTiO_2_N with 2nd photocatalyst Ru/Rh-SrTiO_3_	Simulated AM 1.5 G	0.002 M FeCl_3_	“	60.8	29.2	“

^1^ The units are [μmol g^−1^ h^−1^].

**Table 5:** Reported Ruddlesden–popper perovskites photocatalysts for water splitting.

Perovskite (Band Gap [eV])	Catalyst	Incident Light	Solution	Cocatalyst	H_2_^1^	O_2_^1^	Ref.
La_2_NiO_4_ (1.5)		Xe lamp and a 1.5 AM filter.	0.02 M Na_2_SO_3_ + 0.02 M Na_2_S	Pd	16		[345]
Sr_2_TiO_4_ (3.5)	Cr-doped Sr_2_TiO_4_	500 W Hg lamp, λ > 420 nm	0.05 M Na_2_SO_3_	1 wt.% Pt	180		[334]
	“	500 W Hg lamp	“	“	430		“
	La/Rh co-doped Sr_2_TiO_4_	500 W Hg lamp, λ > 420 nm	0.05 M Na_2_SO_3_	1 wt.% Pt	400		[339]
	La/Fe co-doped Sr_2_TiO_4_	“	“	“	180		[340]
La_2_CuO_4_ (1.3)	Cu_2_O/La_2_CuO_4_	125 W Hg lamp	10 vol.% methanol	-	83		[344]
Sr_3_Ti_2_O_7_ (3.2)	Sr_3_Ti_2_O_7_	400 W Hg lamp	-	1 wt.% NiO	80	42	[335]
Ca_3_Ti_2_O_7_ (3.3)	Rh-doped Ca_3_Ti_2_O_7_	300 W Xe lamp, λ > 420 nm	10 vol.% methanol	0.1 wt.% Pt	3.4		[338]
Sr_4_Ti_3_O_10_ (3.3)	Sr_4_Ti_3_O_10_	400 W Hg lamp	-	NiO_x_	170		[346]
H_2_CaNaNb_3_O_10_ (3.5)	H_2_CaNaNb_3_O_10_	300 W Xe lamp, λ ≥ 300 nm)	0.005 M Fe(NO_3_)_3_ + HNO_3_, (pH = 2)			32	[336]
	“	“	10 vol.% methanol	0.1 wt.% Pt	660		“
HCa_2_Nb_3_O_10_ (3.5)	HCa_2_Nb_3_O_10_	“	0.005 M Fe(NO_3_)_3_ + HNO_3_, (pH = 2)			100	“
K_2_CaNaNb_3_O_10_ (3.3)	K_2_CaNaNb_3_O_10_	“	0.01 M NaIO_3_	0.1 wt.% RuO_2_		110	“
KCa_2_Nb_3_O_10_ (3.5)	KCa_2_Nb_3_O_10_	“	0.01 M NaIO_3_	“		30	“
Sr_3_FeTaO_7_ (2.3)	Sr_3_FeTaO_7_	500 W Hg lamp, λ > 400 nm	0.05 M Na_2_SO_3_	1 wt.% Pt	150		[270]
	“	500 W Hg lamp	“	“	1050		“
LaSrFeO_4_ (2.2)	LaSrFeO_4_	500 W Hg lamp, λ > 400 nm	“	1 wt.% Pt	158		[347]
La_2_SrFe_2_O_7_ (2.1)	La_2_SrFe_2_O_7_	“	“	“	103.2		“
K_2_LaTa_2_O_6_N (2.2)	Ga-doped K_2_LaTa_2_O_6_N	300 W Xe lamp, λ > 420 nm	0.005 M NaI	1 wt.% Pt	92		[337]

^1^ The units are [μmol g^−1^ h^−1^].

**Table 6:** Summary of Aurivillius photocatalytic systems for water splitting.

Perovskite (Band Gap [eV])	Catalyst	Incident Light	Solution	Cocatalyst	H_2_^1^	O_2_^1^	Ref.
Bi_2_WO_6_ (2.9)	Bi_2_WO_6/_BiFeWO_6_	250 W Xe lamp	25 vol.% methanol	-	65		[354]
Bi_2_MoO_6_ (2.6)	Bi_2_MoO_6_ with 2nd photocatalyst Pt/ZnIn_2_S_4_	200 W Xe lamp λ > 420 nm	-	0.5 wt.% CoO_x_	30.8	15.7	[361]
Bi_2_CrO_6_ (1.9)	Bi_2_CrO_6_	300 W Xe lamp λ > 420 nm	0.02 M Na_2_SO_3_, 0.02 M Na_2_S	IrO_2_	14		[362]
	“	“	0.005 M AgNO_3_	IrO_x_		200	“
	Bi_2_CrO_6_ with 2nd photocatalyst Ru/SrTiO_3_:Rh	“	0.002 M FeCl_2_ (pH 2.5)	0.3 wt.% IrO_2_	3.5	1.8	“
Bi_3_TiNbO_9_ (3.2)	Cr/Nb co-doped Bi_3_TiNbO_9_	500 W Hg lamp, λ > 250 nm	0.05 M Na_2_SO_3_	1 wt.% Pt	170		[356]
	W-doped Bi_3_TiNbO_9_	300 W Xe lamp λ > 300 nm	-	Rh, CrO_x_	2000	800	[357]
	Bi_3_TiNbO_9_	“	-	Rh, CrO_x_	140	100	“
	“	300 W Xe lamp	10 vol.% methanol	0.6 wt.% Rh/Cr_2_O_3_	400	200	[358]
	“	“	20 vol.% methanol	Pt	309		[363]
	“	“	2 gL^−1^ AgNO_3_	-		161	“
	Bi_3_TiNbO_9_-Bi_2_MoO_6_	“	-	Rh/CrO_x_	600	300	[359]
Bi_6_Fe_2_Ti_3_O_18_ (2.6)	Bi_6_Fe_2_Ti_3_O_18/_BiOBr	300 W Xe lamp λ > 400 nm	2 gL^−1^ AgNO_3_	-		50	[364]

^1^ The units are [μmol g^−1^ h^−1^].

## Data Availability

No new data were created or analyzed in this study. Data sharing is not applicable to this article.
